# Genome-wide identification and characterization of the GDSL lipase gene family in *Dendrobium catenatum* and their potential role in drought stress tolerance and stomatal outer cuticular ledge formation

**DOI:** 10.3389/fpls.2025.1722133

**Published:** 2025-12-19

**Authors:** Jing Tang, Jiaying Li, Chunyan Tang, Xingyu Han, Haiying Zhang, Beiqi Yang, Long Xiao, Ruiying Li, Hangxing Liu, Dengjin Pi, Qinsong Liu, Disha Hu, Ke Tian, Youfa Li, Qian Wang, Lin Qin

**Affiliations:** 1 Key Laboratory of Oral Disease Research of the Education Department of Guizhou Province, School of Stomatology, Zunyi Medical University, Zunyi, China; 2 Institute of Biomedical Engineering, Kunming Medical University, Kunming, China; 3 Guizhou Engineering Research Center of Industrial Key-technology for Dendrobium Nobile, Zunyi Medical University, Zunyi, China; 4 National Key Laboratory of Crop Genetic Improvement, College of Life Science and Technology, Huazhong Agricultural University, Wuhan, China

**Keywords:** *Dendrobium catenatum*, GDSL lipases, stomatal outer cuticular ledge, expression pattern, subcellular localization, drought tolerance

## Abstract

**Background:**

*Dendrobium catenatum*, a drought-resistant medicinal orchid, exhibits unique adaptations to arid environments; however, the underlying molecular mechanisms remain largely unknow. The formation of the abnormal stomatal outer cuticular ledge (OCL) is prevalent in *D. catenatum* and is thought to contribute to its drought tolerance. Despite this, the GDSL lipases that regulate drought resistance in *D. catenatum* have not yet been identified. This study aimed to systematically identify the GDSL lipase family in *D. catenatum*, analyze their expression patterns, screen for candidates highly expressed in the leaf epidermis and stomatal guard cells, and validate their roles through drought tolerance assays and stomatal OCL characterization.

**Methods:**

A total of 58 GDSL lipase genes were identified from the *D. catenatum* genome. Nine endoplasmic reticulum-localized, drought-responsive candidates were selected for functional characterization in *Arabidopsis*.

**Results:**

Overexpression of *D. catenatum GDSL (DcaGDSL) 25*, *39*, *47*, and *52* in *Arabidopsis* decreased drought tolerance, with *DcaGDSL47*-overexpressing lines exhibiting accelerated water loss. Notably, *DcaGDSL47*, which is enriched in stomatal, reduced drought tolerance, accelerated stomatal water loss, and caused the degradation of stomatal OCL when overexpressed in *Arabidopsis*. These findings suggest that *DcaGDSL47* plays a key role in regulating stomatal OCL formation and drought adaptation.

**Conclusion:**

This study highlights the essential roles of GDSL lipases in modulating stomatal OCL formation and drought adaptation in *D. catenatum*, providing a molecular basis for further investigation of drought resistance mechanisms from the perspective of stomatal OCL formation.

## 1 Introduction

*Dendrobium catenatum* (also called *Dendrobium officinale*), belonging to the *Dendrobium* genus (Orchidaceae), is an epiphytic orchid that grows naturally on the surface of tree bark or rocks, where it undergoes periodic drought stress (Ling et al., 2016; Huang et al., 2020; Xia et al., 2024). To survive harsh environments, *D. catenatum* has developed several drought-adaptative traits, including thicker leaves and cuticles, well-developed succulent pseudobulbs for water storage, facultative crassulacean acid metabolism (CAM) photosynthetic pathways with high water-use efficiency, and abundant polysaccharides (Zhang et al., 2014; Yan et al., 2015; Yang et al., 2016; Zhang et al., 2016; Huang et al., 2020). Thick cuticle is a key water conservation strategy (Yang et al., 2016; Sun et al., 2020). Increased epidermal wax accumulation in leaves can further reduced water loss and improve plant survival under drought stress (Han et al., 2022). The facultative CAM pathway contributes to drought adaptation by facilitating nocturnal carbon dioxide uptake, thereby minimizing daytime water loss (Silvera et al., 2009; Zhang et al., 2014). Under drought conditions, *D. catenatum* exhibits a metabolic shift from C_3_ to CAM photosynthesis (Zhang et al., 2014; Yang et al., 2016). These structural and physiological traits effectively promote epiphytic adaptation to water-deficient environments (Sun et al., 2014; Sun et al., 2019; Sun et al., 2020). Accumulation of compatible solutes (osmolytes), including sugars, sugar alcohols, amino acids, and organic acids, is a key drought resistance strategy. These solutes increase cellular osmotic potential, prevent water loss, and maintain turgor (Zhang et al., 1999; Yan et al., 2015). Water-soluble polysaccharides (WSPs) in *D. catenatum* increase drought tolerance by acting as compatible solutes and facilitating water uptake under osmotic stress (He et al., 2017; Yu et al., 2017; Yu et al., 2019; Yu et al., 2021). During prolonged stress, WSPs stored in pseudobulbs are mobilized to sustain stress tolerance (Stancato et al., 2001; Yang et al., 2016). In addition, the endophytic bacterium *Sphingomonas paucimobilis* ZJSH1 (Li et al., 2023) and orchid mycorrhizal fungi (OMF) isolated from other orchids can promote growth and increase drought tolerance in *D. catenatum* (Li et al., 2021). Furthermore, an observed expansion of subtilisin-like protease genes in the *D. catenatum* genome, which regulate stomatal density and spatial distribution (Berger and Altmann, 2000; Yan et al., 2015), may contribute to its drought resistance. *D. catenatum* is considered a valuable model for elucidating plant drought tolerance mechanisms because of its high drought tolerance (Su and Zhang, 2003; Yan et al., 2015; Zhang et al., 2016; Wan et al., 2019; Huang et al., 2020). Studying how *D. catenatum* responds to and copes with natural drought stresses may provide novel insights into these mechanisms.

*D. catenatum* is a valued traditional Chinese medicinal herb with diverse pharmacological and ornamental properties (Huang et al., 2020). It contains several medicinal components, including polysaccharides, alkaloids, and flavonoids (Zhan et al., 2020, 2021), which exhibit anti-inflammatory, immune-enhancing, antioxidant, antitumor, and hepatoprotective effects (Ng et al., 2012; Xing et al., 2013; Tang et al., 2017; Teixeira da Silva and Ng, 2017; Yu et al., 2021). Cultivation of *D. catenatum* is necessary to increase yield because of its slow growth rate and limited natural distribution. Drought stress severely restricts normal growth, leading to substantial yield losses in cultivated and wild populations (Ramakrishna and Ravishankar, 2011; Ling et al., 2016; Chen et al., 2020b; Zhang et al., 2021, 2023). The stem, which is the primary medicinal part of *D. catenatum*, shows the most significant biomass reduction under drought stress (Han et al., 2022). Water availability considerably influences polysaccharide accumulation (Zhang et al., 2016; Huang et al., 2020). However, the molecular mechanisms underlying drought resistance of *D. catenatum* remain largely unknown. Thus, screening and identifying drought-responsive candidate genes in the *D. catenatum* genome and investigating their functions in stress adaptation is crucial.

Stomata are the primary channels for gas exchange between plants and the external environment, playing key roles in regulating transpiration and water loss, as well as drought tolerance (Nilson and Assmann, 2007; Hunt et al., 2017). A highly cutinized stomatal outer cuticular ledge (OCL) forms around the stomatal pores at the upper edge of the guard cell walls during stomatal development (Willmer and Fricker, 1996; Hunt et al., 2017). Abnormal stomatal OCL formation, characterized by the absence or fusion of the ledge, can disrupt transpiration rates and drought tolerance, while also altering the internal CO_2_ concentration (Ci) and photosynthetic efficiency (Hunt et al., 2017; Tang et al., 2020). For example, *Arabidopsis* glycerol-3-phosphate acyltransferases GPAT4 and GPAT8, which are involved in cutin biosynthesis, demonstrate stomatal OCL deficiency and accelerated water loss in *gpat4*/*gpat8* mutants (Li et al., 2007). The long-chain acyl-CoA synthetase LACS2, which is crucial for cutin synthesis, exhibits stomatal OCL defects and drought sensitivity in *lacs2* mutants (Schnurr et al., 2004; Macgregor et al., 2008). The *cer9* mutant shows a thickened stomatal OCL, decreased transpiration, and improved drought tolerance, owing to elevated C18 cutin monomers and increased C24/C26 fatty acids (Lu et al., 2012). The proline-rich guard cell wall protein FOCL1 is essential for the proper formation of the stomatal OCL. The *focl1* mutant exhibits fused cuticular layers over the stomata, impaired stomatal OCL development, decreased transpiration, and enhanced drought tolerance (Hunt et al., 2017). *Arabidopsis* GDSL lipase OSP1 catalyzes the conversion of very long-chain fatty acyl (VLCFA) -CoAs to VLCFAs and CoA. The *osp1* mutant exhibits decreased wax content, fused stomatal OCL coverage, and improved drought resistance (Tang et al., 2020). Collectively, these findings indicate that stomatal OCL plays a critical role in regulating plant drought adaptation, and its formation is closely linked to structural and compositional modifications of epidermal waxes, cutin, and cell walls. Thus, we hypothesized that the abnormal stomatal OCL formation in *D. catenatum* contributes to its drought tolerance.

GDSL esterases/lipases are lipid hydrolases with a conserved GDSL motif at their N-terminus. They have four invariant catalytic residues—Ser, Gly, Asn, and His in blocks I, II, III, and V, respectively—and are classified as SGNH hydrolases (Akoh et al., 2004). The GDSL esterases/lipases family is extensively found in plants, with 105 members identified in *Arabidopsis* (Lai et al., 2017), 114 in *rice* (Chepyshko et al., 2012), 121 in *Brassica napus* (Dong et al., 2016), 194 in *soybeans* (Su et al., 2020), and 54 in *D. catenatum* (Zhan et al., 2022). These enzymes play crucial roles in plant growth, development, and response to abiotic stress (Shen et al., 2022). Recent studies have highlighted the roles of specific GDSL esterases/lipases in cuticle formation, stomatal development, and drought adaptation (Shen et al., 2022). In rice, WDL1 localizes to the endoplasmic reticulum, where it regulates wax biosynthesis and modulates water loss. Furthermore, *wdl1* mutants exhibit increased transpiration rates, reduced stomatal/epidermal cell sizes, but increased stomatal and epidermal cell density per unit area (Park et al., 2010). In *Arabidopsis*, cuticle-disrupting factor CDEF1 exhibits cutinase activity and induces epidermal defects (Takahashi et al., 2010). Notably, *Arabidopsis* OSP1 is highly expressed in the epidermis and stomatal guard cells, and mediates wax biosynthesis and stomatal OCL formation. The *osp1* mutant exhibits fused cuticular layers that occlude approximately 50% of stomata, resulting in decreased stomatal conductance and transpiration rates while enhancing drought tolerance (Tang et al., 2020). In addition, *Arabidopsis* guard cell-enriched GDSL lipases (GGL7, GGL22, and GGL26) redundantly regulate stomatal dynamics, density, morphology, and plant-water relations (Xiao et al., 2021). Although *D. catenatum* serves as a model species for studying drought survival strategies in highly tolerant herbs (Wan et al., 2019), the GDSL lipases that regulate stomatal OCL formation and drought tolerance in this orchid remain uncharacterized. Therefore, this study aimed to systematically identify the GDSL lipase family in *D. catenatum*, analyze their expression patterns, screen candidates with high expression in the leaf epidermis and stomatal guard cells, and validate their roles via drought tolerance assays and stomatal OCL characterization. Our findings provide a foundation for elucidating the molecular mechanisms underlying stomatal OCL formation and drought resistance in *D. catenatum*.

## 2 Materials and methods

### 2.1 Identification of *D. catenatum* GDSL lipase genes and phylogenetic analyses

A total of 105 *Arabidopsis* GDSL protein sequences were retrieved from The Arabidopsis Information Resource (TAIR) database (https://www.arabidopsis.org/) and queried against the *D. catenatum* genome [taxid:906689] (Zhang et al., 2017) using BLASTP with an E-value cut-off of 1e-10. The retrieved sequences were validated using a hidden Markov model (HMM) search. The GDSL lipase conserved domain HMM profile (PF00657) was obtained from Pfam [http://pfam.xfam.org/] (Chepyshko et al., 2012), and HMMER scanning was conducted with 0.01 as the cutoff value. Genes containing the complete PF00657 domain were verified using the Conserved Domain Database, and the structurally similar PF13472 domain was included as an additional screening criterion. The candidate *D. catenatum* GDSL lipase genes and their corresponding protein IDs were identified. Associated mRNA, protein, and genome sequences, as well as annotation data, were extracted from the *D. catenatum* genome database [PRJNA262478] (Zhang et al., 2016) using TBtools (Chen et al., 2020a). GDSL lipase properties, including amino acid count, theoretical isoelectric points (pIs), and molecular weights (MWs), were examined using ExPASy (https://www.expasy.org), whereas the coding sequence (CDS) length was determined using SnapGene software. The protein sequences of the GDSL lipases from *Arabidopsis* (105), *rice* (114), and *D. catenatum* (58) were aligned with ClustalW using the following parameters: Gap Opening 15, Gap Extension 6.66, and DNA Weight Matrix selection IUB. A phylogenetic tree was constructed using the maximum likelihood method with the Jones-Taylor-Thornton (JTT, 1992) model of amino acid substitution (Jones et al., 1992). The tree with the highest log-likelihood (-27, 941.21) is shown. Branch support values indicate the percentage of replicate trees in the taxa that were clustered together, with replicates determined adaptively (Kumar et al., 2024). The analysis included 287 amino acid sequences. All evolutionary analyses were conducted in MEGA12 using up to 8 parallel threads. The final tree visualization was optimized using the iTOL online tool [https://itol.embl.de/itol.cgi] (Letunic and Bork, 2024).

### 2.2 Conserved motifs, gene structure, and conserved domain analyses

Conserved motifs in the *D. catenatum* GDSL (DcaGDSL) proteins were identified using MEME [https://meme-suite.org/meme/tools/meme] (Bailey et al., 2009) with the following parameters: maximum motifs = 10 and optimum width = 6–50 amino acids. Gene structure analysis was conducted using the online tool GSDS (http://gsds.gao-lab.org) based on the *D. catenatum* genome annotation [gff files] (Hu et al., 2015). TBtools (v2.127) was utilized to integrate the phylogenetic trees, conserved motifs, and gene structures. The conserved domains were analyzed using MEME with the default parameters.

### 2.3 *Cis*-acting regulatory element assays

Putative promoter regions (2.0 kb upstream of the start codon ATG) for *DcaGDSL* genes were extracted from the *D. catenatum* genome using TBtools. Furthermore, the promoter sequences were analyzed for *cis*-acting elements using the PlantCARE online tool (Lescot et al., 2002). The results were processed with Microsoft Excel and visualized with the R package pheatmap (v1.0.13). The final figures were prepared using Adobe Illustrator 2020.

### 2.4 Spatial expression profiles of the *DcaGDSL* genes

The spatial expression profiles of the *DcaGDSL* genes were examined using previously published transcriptome data from *D. catenatum* (Zhang et al., 2017; Wan et al., 2018), encompassing various tissues and drought stress conditions. Heatmaps were generated based on the fragments per kilobase of exon per million mapped fragments (FPKM) values, and visualization and processing were performed using the pheatmap package in R (v3.41).

### 2.5 Plant materials and stress treatments

*D. catenatum* clones were cultivated in transparent plastic pots (10 cm in diameter) using the bark as the matrix. Eight-month-old plants were acclimated for one month in a growth chamber under controlled conditions (12/12 h light/dark cycle; approximately 100 μmol m^-2^ s^-1^ light intensity; 27/25 °C day/night; 80% relative humidity). Uniformly robust plants were selected for experiments. For the drought treatment, plants were irrigated on day 1, and water was withheld for 7 or 14 days. Leaf samples were collected at designated time points, flash-frozen in liquid nitrogen, and stored at 80 °C for *DcaGDSL* gene expression pattern analysis. Three biological replicates were used for each experiment. Samples were collected from the roots, stems, leaves, flower buds, flowers, capsules, sepals, petals, lips, seeds, asymbiotic germination seeds, and protocorms for *DcaGDSL* genes tissue-specific expression analysis.

*Arabidopsis* and *Nicotiana benthamiana* plants were grown in a greenhouse (21 °C; 50–60% relative humidity; 16/8 h light/dark cycle; 80 µmol m^-2^ s^-1^ light intensity).

### 2.6 RNA isolation and qRT-PCR analyses

Total RNA was extracted from *D. catenatum* tissues (including drought-treated leaves at different time points and organs) using an RNAiso Plus Kit (9108, Takara, Beijing), following the manufacturer’s instructions for polysaccharide/polyphenol-rich samples. RNA purity was evaluated with a NanoPhotometer^®^ N60 Touch spectrophotometer (Implen, Munich, Germany). First-strand cDNA was synthesized from 2.0 μg RNA using the PrimeScript™ RT reagent Kit (RR037A, Takara). Gene-specific Premiers for candidate *DcaGDSLs* and reference genes were designed using Primer Premier 5 and validated using NCBI Primer-BLAST. qRT-PCR was conducted on a CFX96™ Real-Time System (Bio-Rad, California, USA) using ChamQ Universal SYBR qPCR Master Mix (Q711, Vazyme, Nanjing). *DcaActin7* served as the internal control. Relative expression under drought stress was calculated versus the 0 h samples (set as 1.0), whereas tissue-specific expression was normalized to the root samples. Data represent the means of three biological replicates, analyzed using Bio-Rad CFX Manager. **Supplementary Table 1** lists all the primers used in this study.

### 2.7 Subcellular localization

Full-length cDNA sequences were cloned into the pEarleyGate101 vector (Earley et al., 2006) to generate *35S: DcaGDSLs-YFP* fusion constructs to determine the subcellular localization of selected *DcaGDSLs* (*DcaGDSL3*, *5*, *6*, *25*, *33*, *35*, *39*, *46*, *47*, and *52*). These constructs were transformed into Agrobacterium GV3101 and co-infiltrated with the endoplasmic reticulum (ER) marker *OFP-HDEL* into *N. benthamiana* leaves. Fluorescence was assessed three days after infiltration using a confocal microscope (STELLARIS 8, Leica, Wetzlar, Germany; excitation/emission: 514/530 nm for YFP and, 552 nm/600 nm for OFP). **Supplementary Table 1** lists all the primers used in this study.

### 2.8 Generation of transgenic plants

Heterologous overexpression involved cloning *DcaGDSL* genes (*DcaGDSL5*, *6*, *25*, *33*, *35*, *39*, *46*, *47*, and *52*) into *pEarleyGate101* to generate *35S:DcaGDSL-YFP* constructs and transformed into *Arabidopsis* (accession Col-0) via a floral dip. Transgenic plants were screened using a 2% glufosinate ammonium solution (A614229, Sangon Biotech, Shanghai) and validated via YFP Fluorescence detection (MF53-N, Mshot, Guangzhou). *DcaGDSL* expression levels were quantified using qRT-PCR as previously described (Tang et al., 2020; Xiao et al., 2021). Two independent T_2_ lines per construct were utilized for the drought stress assays.

### 2.9 Drought stress and water loss analyses

Three-week-old transgenic and Col-0 plants (16 or 25 plants per pot; standardized soil weight and moisture) were subjected to water withholding for 8–9 days after well-watered growth, followed by a 2-day rehydration period. The drought response was photographically documented at three stages: pre-stress, peak stress (when >90% of Col-0 plants exhibited wilting), and recovery. The experiments included three biological replicates and were performed as previously described (Tang et al., 2020; Xiao et al., 2021).

For water loss analyses, transgenic plants and Col-0 leaves were detached from 4-week-old plants grown in a well-controlled greenhouse, dehydrated under laboratory conditions, and weighed using a microbalance at the indicated time points. The water loss rate was calculated as follows: Water loss rate = (initial fresh weight – fresh weight at each time point)/initial fresh weight × 100%.

### 2.10 Expression pattern analyses

The approximately 2.0 kb promoter regions (upstream of ATG) of the selected *DcaGDSLs* (*DcaGDSL25*, *39*, *47*, and *52*) were cloned into pLP100 (Szabados et al., 1995; Charrier et al., 1996). All constructs were verified using Sanger sequencing. *DcaGDSL_pro_::GUS* fusion constructs were transformed into Col-0 via floral dip (Zhang et al., 2006) to drive β-glucuronidase (GUS) reporter expression. Transformants were selected using 50 mg/L kanamycin and validated via GUS staining. Furthermore, 14-day-old seedlings following germination were grown on 1/2 Murashige and Skoog medium, and 3-week-old plant leaves were incubated in GUS staining buffer (SL7160, Coolaber, Beijing) at 37 °C for 8 h, destained in 80% ethanol, and imaged using a stereomicroscope (TS100, Nikon, Beijing). Three independent transgenic lines were analyzed, and representative lines were photographed.

### 2.11 SEM, stomatal density, and stomatal morphology analyses

For scanning electron microscopy (SEM) analysis, leaf segments of *D. catenatum* (2 × 2 mm) and the seventh or eighth rosette leaves of 4-week-old *DcaGDSL47*-overexpressing *Arabidopsis* were collected from at least five randomly selected plantlets per genotype. Samples were fixed in 2.5% glutaraldehyde in 0.1 M phosphate buffer (pH 7.4) for 24 h at 4 °C, rinsed with 0.1 M phosphate buffer four times for 15 min each, and post-fixed in 1% osmium tetroxide for 1.5 h at 21 °C. After rinsing four times with 0.1 M phosphate buffer for 10 min each, the samples were dehydrated through a graded ethanol series (30, 50, 70, 80, 90, and 100%) and dried using a critical point dryer (EM CPD300, Leica) with liquid CO_2_. The dried samples were sputter-coated with 25–30 nm gold palladium (EM ACE600, Leica), and the central areas derived from the leaf abaxial epidermal layer were imaged using a scanning electron microscope (SU8010, Hitachi, Tokyo, Japan) at an accelerating voltage of 3.0 kV. For stomatal density and morphology analyses, stomata numbers were counted using ImageJ software. Stomatal pore length and width, and stomatal complex length and width were measured using ImageJ.

## 3 Results

### 3.1 Stomatal OCL formation correlates with drought tolerance in *Dendrobium*

**Figure 1:**
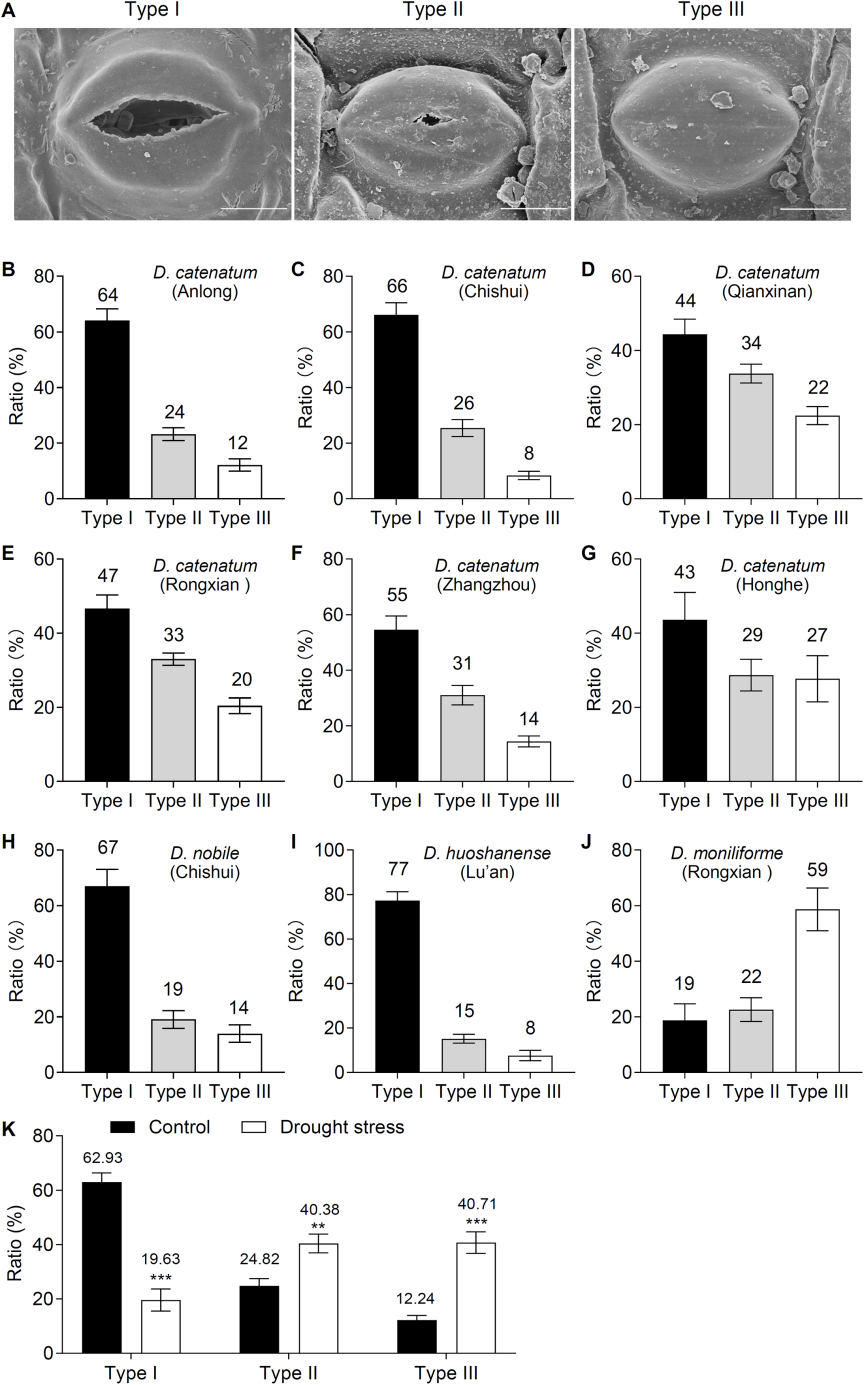
Scanning electron microscopy (SEM) analyses of the stomatal outer cuticular ledge (OCL) morphology in *Dendrobium* species across geographic regions. **(A)** SEM analyses reveal the stomatal pore types in *D*. *catenatum*. Type I, normal opened stomatal pores; Type II, partially occluded pores; and Type III, fully occluded pores. Scale bars = 10 µm. **(B–G)** The ratio of Type I, Type II, and Type III stomata in *D*. *catenatum* from Guizhou (Anlong, Chishui, and Qianxinan), Guangxi (Yulin Rongxian), Fujian (Zhangzhou), and Yunnan (Honghe). **(H–J)** The ratio of the stomatal types in *D*. *nobile* (Chishui, Guizhou), *D*. *huoshanense* (Lu’an, Anhui), and *D*. *moniliforme* (Yulin Rongxian, Guangxi). Values represent the means ± standard error (n = 5, each with at least 80 stomata analyzed). **(K)** The ratio of Type I, Type II, and Type III stomata in *D*. *catenatum* under a 14-day drought stress condition. Values represent the means ± standard error (n = 8, each with at least 110 stomata analyzed). **, *P* < 0.01; ***, *P* < 0.001; Two-tailed unpaired Student’s *t*-test.

*D. catenatum* shows exceptional drought tolerance and can survive in arid environments. We analyzed stomatal OCL morphology in *D. catenatum* leaves from six regions (Anlong, Chishui, and Qianxinan in the Guizhou Province; Yulin Rongxian in Guangxi; Zhangzhou in Fujian; Honghe in Yunnan) using SEM to investigate whether this trait is associated with drought tolerance. Three stomatal types were identified: Type I (normally opened stomata), Type II (partially occluded stomata), and Type III (fully occluded stomata) (**Figure 1A**). Their proportions varied considerably across regions. Type I stomata accounted for 64, 66, 44, 47, 55, and 43% of the respective populations (**Figures 1B–G**), with the highest frequency in Chishui (66%; **Figure 1C**) and the lowest in Qianxinan and Honghe (44% and 43%, respectively; **Figures 1D, G**). Type II stomata ranged from 24 to 34%, peaking in Qianxinan (34%; **Figure 1D**). Type III stomata constituted 12, 8, 22, 20, 14, and 27% of the population, with the highest prevalence in Honghe (27%; **Figure 1G**). Thus, partial stomatal OCL malformation is a universal phenomenon in *D. catenatum* and is influenced by regional environmental factors, suggesting a potential association between stomatal OCL dynamics and drought adaptation.

Additionally, aberrant stomatal OCL formation was observed in other *Dendrobium* species, including *Dendrobium nobile* Lindl. (*D. nobile*, Chishui, Guizhou), *Dendrobium huoshanense* (*D. huoshanense*, Lu’an, Anhui), and *Dendrobium moniliforme* (*D. moniliforme*, Yulin Rongxian, Guangxi) (**Figures 1H–J**). Type I stomatal frequencies were 67, 77, and 19%, respectively, and were the highest in *D. huoshanense* (77%; **Figure 1I**). Type II frequencies (19, 15, and 22%) peaked in *D. moniliforme* (22%; **Figure 1J**), whereas Type III frequencies (14, 8, and 59%) were maximal in *D. moniliforme* (59%; **Figure 1J**), in contrast to *D. huoshanense* minimal abnormality (8%; **Figure 1I**). Thus, *D. moniliforme* showed the highest frequency of stomatal OCL malformation, whereas *D. huoshanense* exhibited the lowest. Collectively, partial stomatal OCL malformations are a widespread phenomenon across the *Dendrobium* genus, albeit with substantial interspecific variation in the stomatal OCL abnormality rates.

We subjected *D. catenatum* to drought stress and analyzed stomatal OCL morphology using SEM to explore the relationship between stomatal OCL formation and drought adaptation. After 14 days of drought treatment, the normally opened stomata (Type I) decreased from 62.93 to 19.63%, whereas partially occluded stomata (Type II) increased from 24.82% to 40.38%, and fully occluded stomata (Type III) increased from 12.24 to 40.71% (**Figure 1K**). Thus, drought-induced shifts to fully occluded stomata (Type III) lead to a decrease in transpiration rates and enhance drought tolerance, explaining *D. catenatum’s* tolerance to arid habitats.

### 3.2 Identification of *D. catenatum* GDSL lipase genes and phylogenetic analysis

*Arabidopsis* GDSL lipase OSP1 regulates stomatal OCL formation and drought tolerance; however, the role of GDSL homologs in *D. catenatum* remains elusive. We queried the *D. catenatum* genomic database on NCBI (taxid: 906689) using *Arabidopsis* GDSL sequences from the TAIR database to identify *D. catenatum* GDSL lipases. After removing redundant/incomplete sequences and verifying the conserved GDSL domain (PF00657) using the Pfam database, 58 *DcaGDSL* lipase genes, designated *DcaGDSL1* to *DcaGDSL58*, were identified (**Supplementary Table 2**). Most *DcaGDSL* genes had a single transcript, except for *DcaGDSL14* (eight transcripts) and *DcaGDSL25*, *32*, *37*, and *57* (two transcripts each). The deduced DcaGDSL lipases range in length from 162 amino acids (aa; DcaGDSL57) to 801 aa (DcaGDSL7), MWs ranging from 17.47 kDa (DcaGDSL57b) to 63.79 kDa (DcaGDSL9), and the pI spanning 4.53 (DcaGDSL35) to 9.78 (DcaGDSL23).

**Figure 2:**
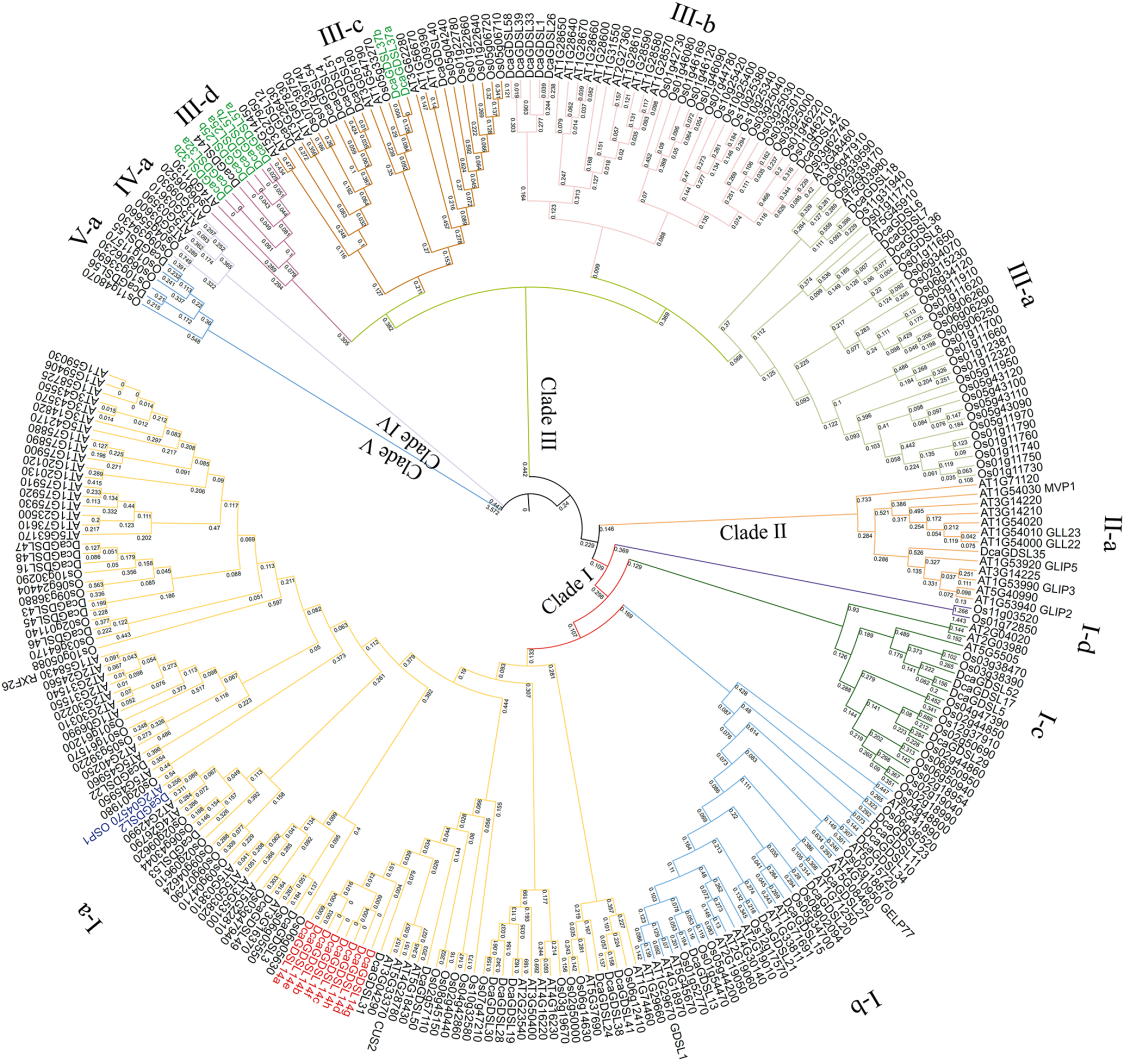
Phylogenetic analysis of the GDSL lipases in *Arabidopsis* (At, 105), *D. catenatum* (Dca, 58), and *rice* (Os, 114). A total of 277 GDSL lipases were utilized to construct the unrooted maximum likelihood phylogenetic tree. The GDSL lipase family gene is divided into five subgroups/clade (I–V), subgroup I comprises four subclades (I-a, I-b, I-c, I-d), subgroup III comprises four subclades (III-a, III-b, III-c, III-d), subgroup II, IV, and V comprises one clade (II-a, IV-a, V-a, respectively). DcaGDSL14 has eight transcripts, which are indicated in red. DcaGDSL25, 32, 37, and 57 have two transcripts, each indicated in green.

We constructed an ML phylogenetic tree using the protein sequences from *D. catenatum*, *Arabidopsis*, and *rice* to investigate the evolutionary relationships between GDSL lipases in *D.* catenatum and other species. We divided the 58 DcaGDSL lipases into 5 subgroups based on their phylogenetic tree (**Figure 2**). *D. catenatum* GDSL lipases (total 58) included 33 members in subgroup I, 1 member in subgroup II, 22 members in subgroup III, and 2 members in subgroup V (57%, 2%, 38%, and 3% respectively). In *Arabidopsis* (total 104), 64 (62%), 12 (11%), 24 (23%), and 4 (4%) GDSL lipases were present in subgroups I–IV, respectively. Whereas *rice* (total 114) had 52 (46%), 56 (49%), 2 (2%), and 4 (3%) GDSL lipases in subgroup I and subgroup III–V, respectively. Conserved clade distributions across species suggest evolutionary preservation of the GDSL functions in plants.

### 3.3 *DcaGDSLs* gene structures and conservative domain

**Figure 3:**
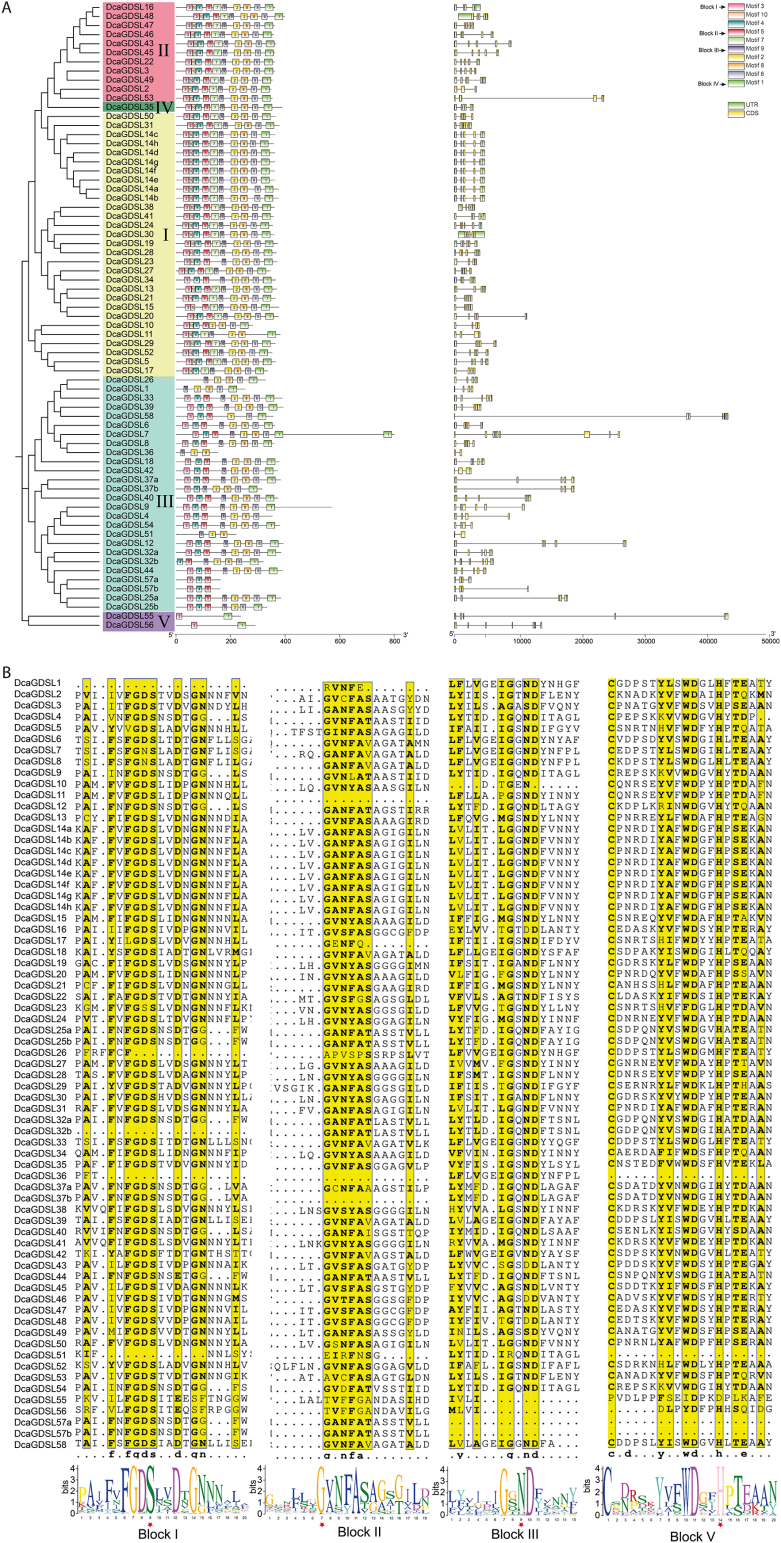
Conserved motifs, gene structures, and conserved domain analyses of the *DcaGDSL* genes. **(A)** Left, A phylogenetic tree was constructed with the Maximum Likelihood method. The different subgroups are indicated with various background colors and letters. Middle, Conserved motifs. Different motifs are represented by various colored boxes and numbers. Right, gene structures. Exon(s), intron(s), and UTR(s) are represented by yellow boxes, black lines, and green boxes, respectively. The phylogenetic tree, conserved motifs, and gene structures were predicted using TBtools. **(B)** Conserved domain architecture of the DcaGDSL proteins. Sequences aligned with DNAMAN7. Conserved domains of the DcaGDSL proteins were examined using the online MEME software. Four blocks in the SGNH-hydrolase family (Blocks I, II, III, and V) are indicated in the background yellow boxes. The motif logo and red stars indicate the conserved residues Ser, Gly, Asn, and His in the four conserved Blocks I, II, III, and V, respectively.

Analysis of the conserved domains in DcaGDSL lipases provides insights into their functional potential. We examined the conserved motifs of 58 DcaGDSL proteins using online MEME software and identified 10 conserved motifs (Motifs 1–10) across 58 DcaGDSL lipases (**Figure 3A**). The motif distribution patterns were clade-specific, implying evolutionary conservation. Members of the subgroups II and IV all contain Motifs 1–10. Notably, Motifs 7 and 10 existed in the subgroups I, II, and IV, whereas DcaGDSL55 and DcaGDSL56 in subgroup V only contain Motifs 1 and 3 (**Figure 3A**). Distinct motif combinations and arrangements across clades suggest functional diversification within this gene family.

The intron-exon structures of the *DcaGDSL* genes were analyzed using GSDS. Phylogenetically related genes showed similar structures, with subgroups III and V having the longest introns, whereas subgroups I and IV had the shortest introns. Additionally, variable distributions of the CDS and UTR regions were observed (**Figure 3A**). The complex structures of the genes make their functions more variable.

Conserved domain analysis via MEME showed that all 58 DcaGDSL lipases contain Blocks I–IV, except for 5 subgroup III members with specific deletions: DcaGDSL1 lacks Block I, DcaGDSL12 and DcaGDSL36 lack Block II, DcaGDSL57 lacks Blocks III and IV, and DcaGDSL36 and DcaGDSL51 lack Block IV(**Figure 3B**). Blocks I–IV include four conserved amino acids, Ser, Gly, Asn, and His, respectively, with Ser serving as the enzymatic core of the DcaGDSL lipases (**Figure 3B**). DcaGDSL1, 26, 36, and 51 in subgroup III lack Ser and Gly. Additionally, DcaGDSL12, 53, 55, and 56 lack Gly. DcaGDSL3, 16, 43, 46, 48, and 49 in subgroup II lack Asn, and DcaGDSL55–DcaGDSL57 also lack Asn. Furthermore, DcaGDSL36, 51, 55, and 57 lack His (**Figure 3B**). The complex protein structures suggest that DcaGDSL lipases, particularly those in subgroup III, exhibit distinct substrate specificities and functional diversities.

### 3.4 *Cis*-acting elements in *DcaGDSL* promoters

**Figure 4:**
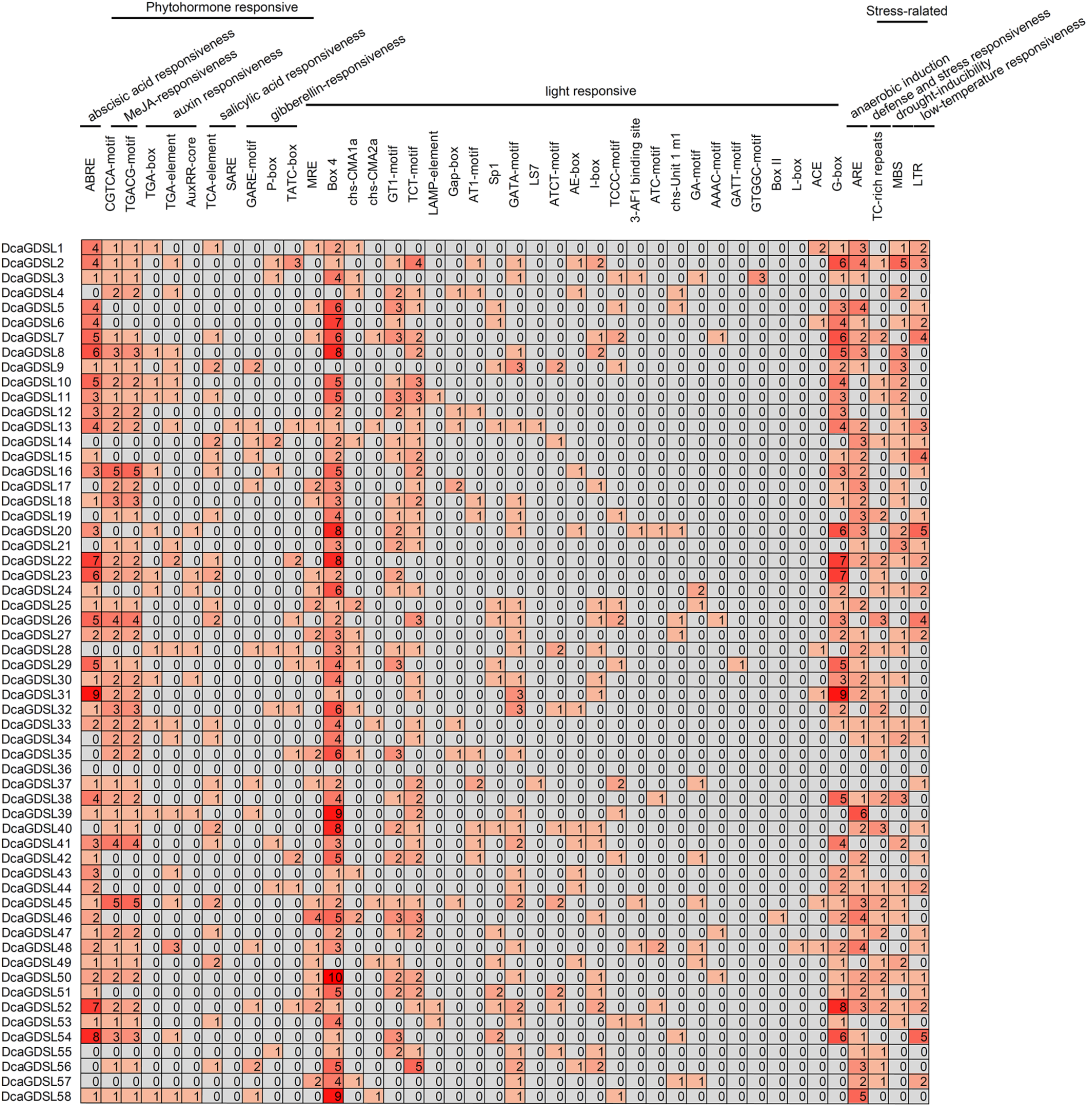
*Cis*-regulatory elements in the *DcaGDSL* promoter regions. Heatmap depicting the number and type of *cis*-elements within the 2.0-kb upstream promoter regions of the *DcaGDSL* genes. Color intensity and grid values indicate the element counts. Analysis performed using PlantCARE.

We analyzed the 2-kb promoter regions upstream of the coding sequences using PlantCARE to investigate the transcriptional regulation of *DcaGDSL* genes. Three functional categories of *cis*-acting regulatory elements (CREs) exist, including phytohormone-responsive, light-responsive, and stress-inducible. Hormone-related elements include the abscisic acid responsive element (ABRE), methyl jasmonate-responsive elements (CGTCA-motif and TGACG-motif), auxin-responsive elements (TGA-box, TGA-element, and AuxRR-core), salicylic acid responsive elements (SARE and TCA-element), and gibberellin-responsive elements (GARE-motif, P-box, and TATC-box). Among them, ABRE- (45 promoters) and MeJA-responsive motifs (CGTCA/TGACG; 43 promoters) exhibited the highest frequencies. The light-responsive elements included MRE, Box 4, GT1-motif, TCT-motif, GATA-motif, and G-box. Box 4 (54 promoters) and G-box (41 promoters) showed the highest frequencies, whereas the GATT-motif (*DcaGDSL29*), Box II (*DcaGDSL46*), and L-box (*DcaGDSL48*) demonstrated promoter-specific occurrences. Stress-related elements included anaerobic induction (ARE), defense/stress response (TC-rich repeats), drought-inducible MYB-binding sites (MBS), and low-temperature response (LTR). Distribution analysis showed that ARE was enriched in 45 *DcaGDSL* promoters, MBS was detected in 33 *DcaGDSL* promoters (*DcaGDSL2* contains 5 MBS copies), LTR was contained in 29 promoters, and TC-rich repeats were contained in 30 promoters. MBS prevalence, particularly in *DcaGDSL2*, suggests its potential role in *D. catenatum* drought tolerance via MYB transcription factor regulation. LTR enrichment suggests roles in cold adaptation (**Figure 4**).

### 3.5 *DcaGDSL* genes spatial expression profiles

Re-analysis of the Zhang et al. (2017) transcriptome data revealed tissue-specific expression patterns of *DcaGDSL* genes across six tissues: green root tips, white part of roots, stems, leaves, flower buds, and sepals (**Supplementary Figure 1**). Although all genes were ubiquitously expressed, spatial divergence was evident. *DcaGDSL1*, *2*, *7*, *17*, *18*, *28*, *29*, *30*, *36*, *38*, and *54* (11 genes) were highly expressed in the roots, and *DcaGDSL16*, *19*, *24*, *37*, *41*, *46*, and *48* (7 genes) were highly expressed in the root tips. *DcaGDSL12*, *43*, and *56* were highly expressed in the stems. *DcaGDSL25*, *26*, *33*, *35*, *39*, *47*, *52*, *55*, *57*, and *58* (10 genes) were highly expressed in leaves, implying that these genes may be highly expressed in stomatal guard cells, especially *DcaGDSL47* and *DcaGDSL58*. *DcaGDSL5*, *6*, *13*, *14*, *15*, *20*, *21*, *22*, *23*, *27*, *31*, *34*, *40*, *42*, *45*, *49*, *50*, *51*, and *53* (19 genes) were highly expressed in the flower buds. *DcaGDSL3*, *4*, *8*, *9*, *10*, *11*, *12*, *32*, and *44* (eight genes) were highly expressed in the sepals. These organ-enriched expression patterns indicate their putative roles in tissue development and environmental sensing.

We analyzed its expression profiles under drought stress using transcription data to investigate *DcaGDSL* functions in *D. catenatum* drought tolerance (Wan et al., 2018). Five genes (*DcaGDSL3*, *5*, *6*, *39*, and *52*) showed substantial down-regulation in the leaves under severe drought stress. However, the expression levels of these five *DcaGDSL* genes in leaves were similar under well-watered and moderate drought stress conditions. Conversely, *DcaGDSL46* was up-regulated under severe drought conditions (**Supplementary Table 3**). Thus, these six genes (*DcaGDSL3*, *5*, *6*, *39*, and *52*) are the candidate regulators of *D. catenatum* drought adaptation.

**Figure 5:**
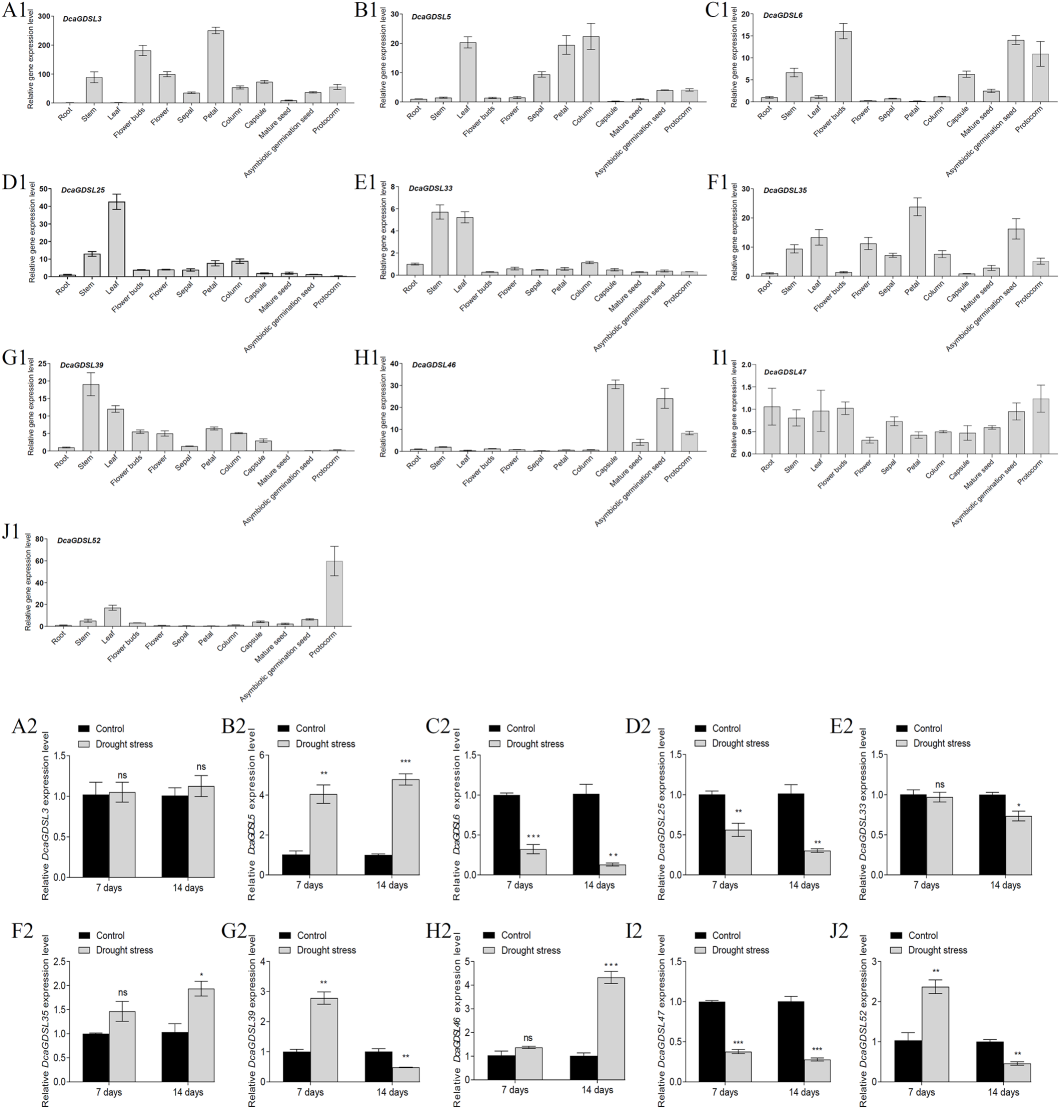
Expression profiles of the *DcaGDSL* genes. **(A1–J1)** Tissue-specific expression of the *DcaGDSL* genes relative to *DcaActin7* revealed by qRT-PCR. The mean expression value was calculated from three independent biological replicates relative to the root expression. **(A2–J2)** Drought-responsive expression of the *DcaGDSL* genes relative to *DcaActin7* in leaves determined via qRT-PCR. Values are represented as means ± standard error (n = 3). *, *P* < 0. 05; **, *P* < 0. 01; ***, *P* < 0. 001; ns, no significant difference; Two-tailed unpaired Student’s *t*-test.

We selected 10 *DcaGDSL* genes (*DcaGDSL3*, *5*, *6*, *25*, *33*, *35*, *39*, *46*, *47*, and *52*) based on high leaf expression or drought responsiveness to identify stomata-enriched DcaGDSL lipases linked to drought tolerance in *D. catenatum*. Spatial expression profiles were examined via qRT-PCR across 12 tissues (roots, stems, leaves, flower buds, flowers, sepals, petals, columns, capsules, mature seeds, asymbiotic germination seeds, and protocorms), revealing that *DcaGDSL3* peaks in the flower buds and petals, suggesting its potential role in floral development (**Figure 5A1**). *DcaGDSL5* was enriched in the leaves, petals, and columns (**Figure 5B1**). Furthermore, *DcaGDSL6* was elevated in the flower buds, asymbiotic germination seeds, and protocorms, and its expression in asymbiotic germination seeds substantially exceeded that in the mature seeds and protocorms, indicating its role in seed asymbiotic germination (**Figure 5C1**). *DcaGDSL25*, *DcaGDSL33*, and *DcaGDSL39* were enriched in the stems and leaves (**Figures 5D1, E1, G1**). *DcaGDSL35* expression was high in the leaves, petals, and asymbiotic germination seeds. Similar to that of *DcaGDSL6*, *DcaGDSL35* expression in asymbiotic germination seeds surpassed that in mature seeds and protocorms, suggesting a functional overlap (**Figure 5F1**). *DcaGDSL46* was enriched in the capsules and asymbiotic germination seeds, with substantially higher expression in the asymbiotic germination seeds than in mature seeds, indicating its involvement in seed development and asymbiotic germination (**Figure 5H1**). *DcaGDSL47* was constitutively expressed (**Figure 5I1**). *DcaGDSL52* was predominantly expressed in the protocorms and leaves, with protocorm expression considerably exceeding that in the other tissues, underscoring its potential role in protocorm development (**Figure 5J1**). These tissue-specific patterns highlight the potential roles of *DcaGDSL* genes in developmental regulation.

Nine-month-old *D. catenatum* plants were subjected to 7- and 14-day drought treatments to characterize the drought-responsive expression of 10 *DcaGDSL* genes. The qRT-PCR analysis revealed no significant changes in *DcaGDSL3* expression under drought stress (**Figure 5A2**). *DcaGDSL5*, *35*, and *46* were significantly up-regulated after drought treatment (**Figures 5B2, F2, H2**). Conversely, *DcaGDSL6*, *25*, *33*, and *47* were down-regulated by drought stress (**Figures 5C2, D2, E2, I2**). *DcaGDSL39* and *DcaGDSL52* were induced at 7 days but were down-regulated after 14 days of drought exposure (**Figures 5G2, J2**). Consistent with the transcriptomic data (Wan et al., 2018), *DcaGDSL6*, *DcaGDSL39*, and *DcaGDSL52* were down-regulated, whereas *DcaGDSL46* was up-regulated after 14 days of drought stress. Overall, nine *DcaGDSL* genes display dynamic, tissue-specific drought responsiveness and represent priority candidates in *D. catenatum* drought adaptation.

### 3.6 Subcellular localization of DcaGDSLs in *N. benthamiana*

**Figure 6:**
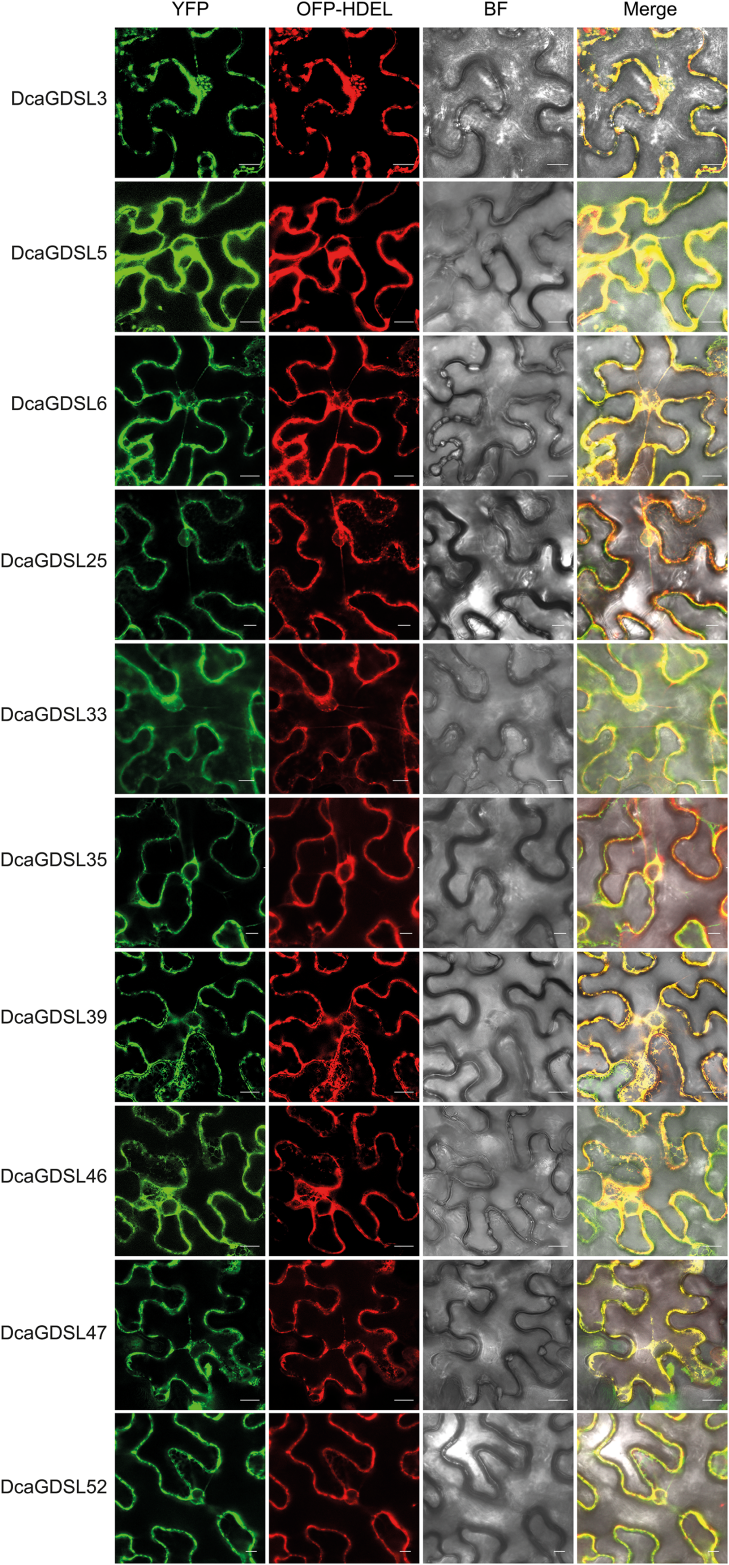
Subcellular localization of the DcaGDSL lipases in the *N. benthamiana* leaf epidermal cells. The endoplasmic reticulum (ER) marker OFP-HDEL was co-infiltrated. Fluorescence observed via confocal microscopy. Scale bars = 10 μm.

The subcellular localization of the 10 selected DcaGDSL proteins was determined by transiently expressing *35S_pro_: DcaGDSL-YFP* fusions in the *N. benthamiana* leaf epidermis. Confocal microscopy show the co-localization of nine proteins (DcaGDSL3, DcaGDSL5, DcaGDSL6, DcaGDSL25, DcaGDSL35, DcaGDSL39, DcaGDSL46, DcaGDSL47, and DcaGDSL52) with the ER marker OFP-HDEL. DcaGDSL33 reveals dual localization in the cytosol and ER, indicating distinct trafficking mechanisms (**Figure 6**). These results demonstrate that the 10 selected DcaGDSLs are predominantly localized in the ER for most DcaGDSL lipases.

### 3.7 Overexpression of *DcaGDSLs* decreases drought tolerance in *Arabidopsis*

**Figure 7:**
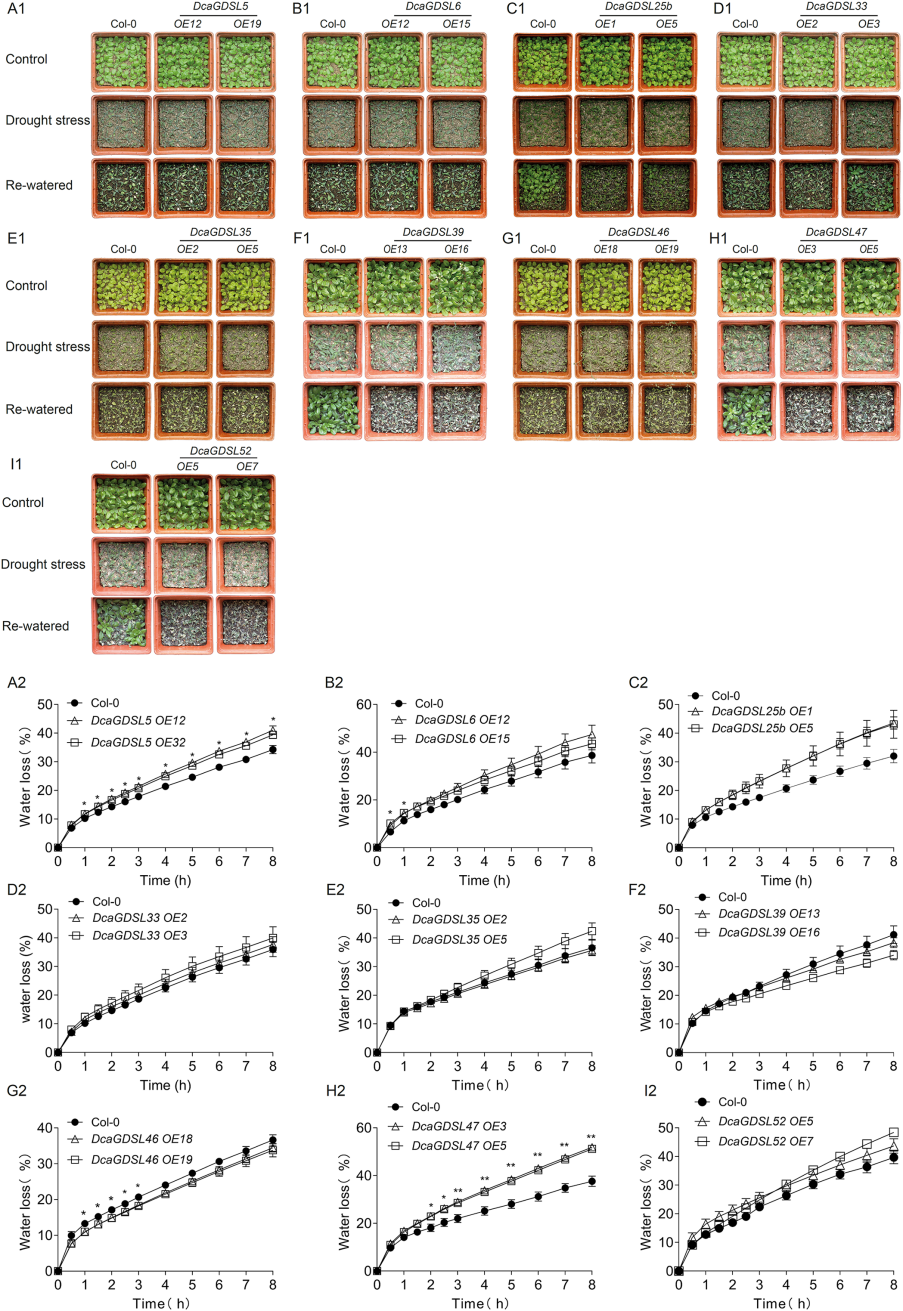
Drought performance and water loss analyses in *DcaGDSL* transgenic *Arabidopsis*. **(A1–I1)** Drought performance of the Col-0 and two transgenic lines expressing *35S_pro_: DcaGDSL5-YFP* (*OE12*, *OE19*) **(A1)**, expressing *35S_pro_: DcaGDSL6-YFP* (*OE12*, *OE15*) **(B1)**, expressing *35S_pro_: DcaGDSL25b-YFP* (*OE1*, *OE5*) **(C1)**, expressing *35S_pro_: DcaGDSL33-YFP* (*OE2*, *OE3*) **(D1)**, expressing *35S_pro_: DcaGDSL35-YFP* (*OE2*, *OE5*) **(E1)**, expressing *35S_pro_: DcaGDSL39-YFP* (*OE13*, *OE16*) **(F1)**, expressing *35S_pro_: DcaGDSL46-YFP* (*OE18*, *OE19*) **(G1)**, expressing *35S_pro_: DcaGDSL47-YFP* (*OE3*, *OE5*) **(H1)**, and expressing *35S_pro_: DcaGDSL52-YFP* (*OE5*, *OE7*) **(I1)**. Three-week-old plants were subjected to drought stress for 8 days, followed by 2 days of re-watering. Images were taken at the indicated time points. Experiments were repeated thrice. **(A2–I2)** Water loss rate in the detached leaves of Col-0 and two transgenic lines, *DcaGDSL5* (*OE12*, *OE9*) **(A2)**, *DcaGDSL6* (*OE12*, *OE15*) **(B2)**, *DcaGDSL25b* (*OE1*, *OE5*) **(C2)**, *DcaGDSL33* (*OE2*, *OE3*) **(D2)**, *DcaGDSL35* (*OE2*, *OE5*) **(E2)**, *DcaGDSL39* (*OE13*, *OE16*) **(F2)**, *DcaGDSL46* (*OE18*, *OE19*) **(G2)**, *DcaGDSL47* (*OE3*, *OE5*) **(H2)**, and *DcaGDSL52* (*OE5*, *OE7*) **(I2)**. Values are means ± standard error (n = 3). *, *P* < 0. 05; **, *P* < 0. 01; Ordinary one-way ANOVA with Bonferroni’s multiple comparisons test.

*DcaGDSL3* was excluded from the functional analysis due to its reproductive organ-specific and drought-insensitive profile in *D. catenatum* leaves. The remaining nine *DcaGDSL* genes were heterologously expressed in *Arabidopsis* via *35S_pro_: DcaGDSLs-YFP* constructs (**Supplementary Figure 2**). Drought tolerance assays showed decreased drought tolerance in the *DcaGDSL25*/*39*/*47*/*52*-overexpressing lines (**Figures 7C1, F1, H1, I1**); whereas, *DcaGDSL5*/*6*/*33*/*35/46-*overexpressing lines demonstrated no substantial difference in drought tolerance compared to Col-0 (**Figures 7A1, B1, D1, E1, G1**). Detached-leaf water loss analyses revealed that *DcaGDSL5/47*-overexpressing lines exhibited accelerated dehydration and enhanced water loss under light conditions (**Figures 7A2, H2**); whereas, *DcaGDSL6/25/33/35/39/46/52*-overexpressing lines showed no significant difference in water loss compared to Col-0 (**Figures 7B2, C2, D2, E2, F2, G2, I2**). Thus, *DcaGDSL25*, *39*, *47*, and *52* are likely to negatively regulate drought tolerance. Moreover, as *DcaGDSL47*-overexpressing lines demonstrated no substantial difference in water loss compared to Col-0 under dark conditions (**Supplementary Figure 3**), *DcaGDSL47* negatively controls drought tolerance via the modulation of stomatal water loss. *DcaGDSL47* (Dca016600) exhibits confirmed lipase activity and correlates with 24 lipid species (Zhan et al., 2022), suggesting its potential role in regulating stomatal dynamics through lipid metabolism. These results suggest that DcaGDSL25/39/47/52 play a key role in regulating drought adaptation.

### 3.8 Tissue-specific expression patterns of *DcaGDSLs*

**Figure 8:**
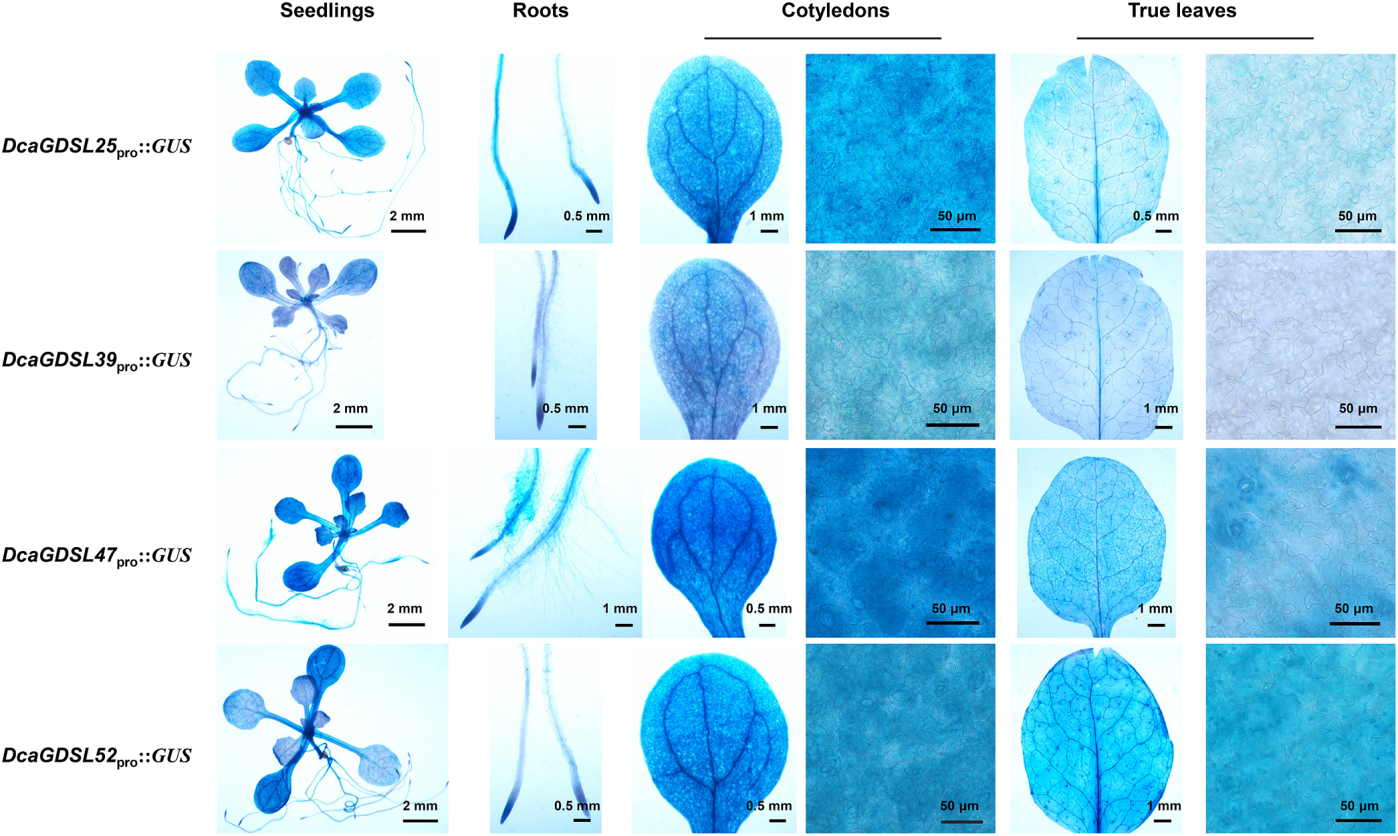
Expression pattern of the *DcaGDSL* genes. Histochemical detection of the GUS expression driven by the *DcaGDSL* promoter in transgenic seedlings, roots, cotyledons, and true leaves of *Arabidopsis*. Images illustrate the representative expression patterns.

More than 95% of the plant water loss occurs via stomatal transpiration, which is a key determinant of drought tolerance. The cuticular composition further modulates drought adaptation by inducing stomatal OCL malformation, thereby changing transpiration rates (Tang et al., 2020). Heterologous overexpression of *DcaGDSL25*, *39*, *47*, and *52* in *Arabidopsis* conferred drought-sensitive phenotypes, suggesting their involvement in stomatal or cuticular regulation. We assessed cell type-specific expression by cloning 2 kb native promoters of *DcaGDSL25*/*39*/*47*/*52* into pPLP100 (Xiao et al., 2021) to drive GUS reporter expression in *Arabidopsis*. GUS staining in three independent *Arabidopsis* lines showed constitutive expression throughout seedlings, with enrichment in the root tips, cotyledon veins, and cotyledon epidermal and guard cells. Notably, *DcaGDSL25/47* exhibited stronger expression in seedlings, roots, and cotyledons than *DcaGDSL39/52* (**Figure 8**). These spatial patterns support their putative roles in the stomatal/cuticular regulation of water loss.

The guard cell expression of *DcaGDSL25*/*39*/*47*/*52* in 3-week-old *Arabidopsis* true leaves was analyzed. GUS staining showed that *DcaGDSL25* was enriched in veins and trichomes, but its expression was weaker in epidermal and guard cells than in cotyledons. *DcaGDSL39* was restricted to the veins and trichomes and was undetectable in the epidermal and guard cells of true leaves. *DcaGDSL52* was strongly expressed in the veins, trichomes, epidermis, and guard cells, exceeding the expression levels of *DcaGDSL25*. These expression patterns implicate the roles of *DcaGDSL25*, *39*, and *52* in trichome and vasculature development, with *DcaGDSL25/52* potentially regulating epidermal and stomatal functions. Notably, *DcaGDSL47* revealed high expression in the trichomes and vasculature, with preferential localization in the guard cells of true leaves (**Figure 8**). Furthermore, this finding aligns with the extreme drought sensitivity observed in *DcaGDSL47*-overexpressing *Arabidopsis*, as well as accelerated water loss in detached leaves under light (peaking at 30 min), and no difference in water loss under dark-induced stomatal closure (**Supplementary Figure 3**). This confirms that *DcaGDSL47* regulates stomatal water loss, likely by modulating stomatal movement and development, including stomatal OCL formation.

### 3.9 DcaGDSL47 modulates stomatal OCL formation

**Figure 9:**
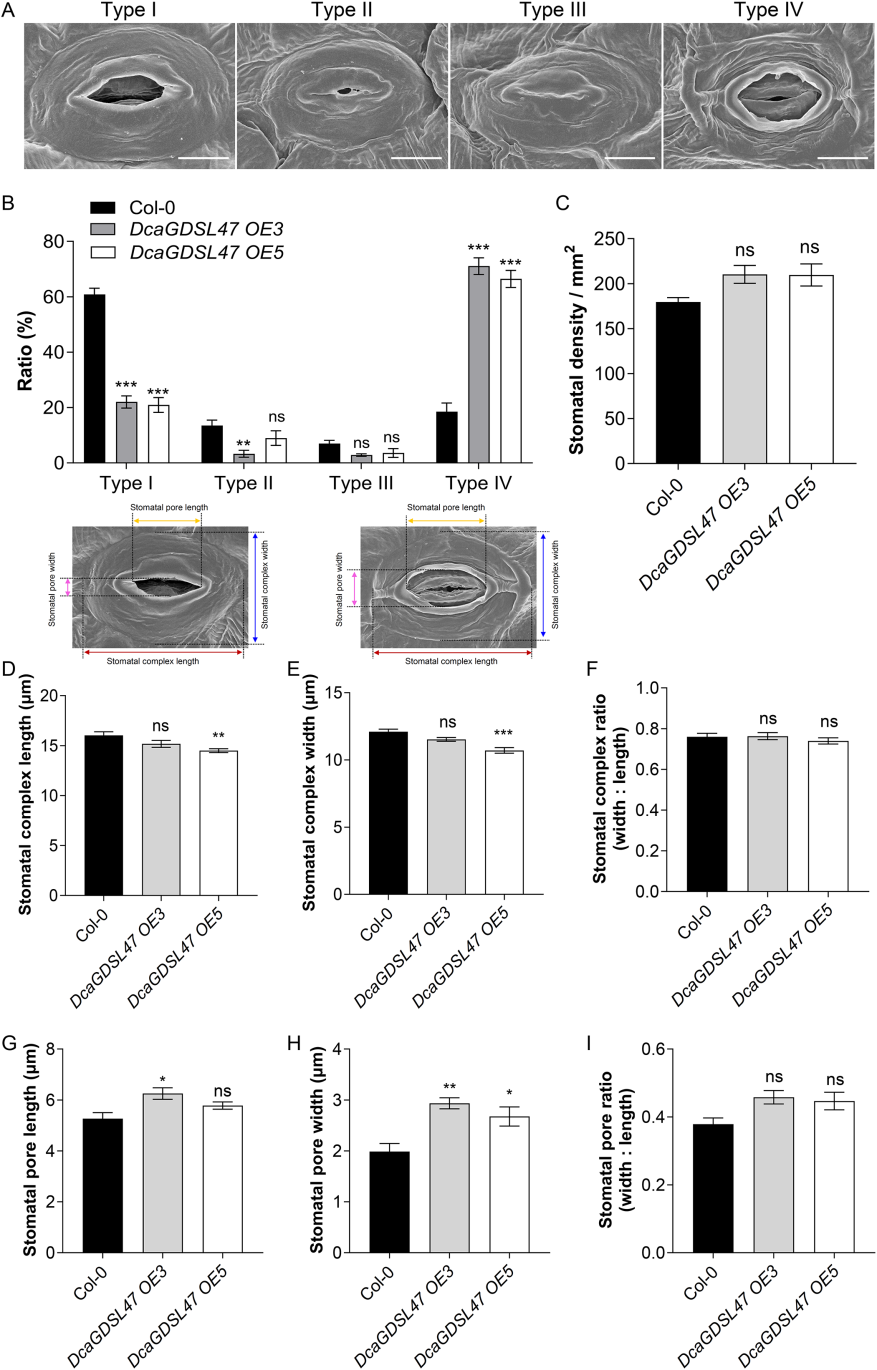
*DcaGDSL47* causes degradation of stomatal outer cuticular ledges (OCL) in *Arabidopsis*. **(A)** SEM analyses show the types of mature leaf stomatal pores. Type I, normal opened stomatal pores; Type II, stomatal pores not fully occluded; Type III, stomatal pores fully covered; Type IV, stomatal OCL partially degraded. Scale bars = 5 μm. **(B)** The ratio of Type I, Type II, Type III, and Type IV stomata in the Col-0 and transgenic lines expressing *35S_pro_: DcaGDSL47-YFP* (*DcaGDSL47 OE3* and *DcaGDSL47 OE5*). Values are means ± standard error (n = 5, each with at least 50 stomata analyzed). **, *P* < 0.01; ***, *P* < 0.001; ns, no significant difference; student’s *t*-test. **(C)** Stomatal density in the abaxial leaves of Col-0, *DcaGDSL47 OE3*, and *DcaGDSL47 OE5*. Values are means ± standard error (n = 5, each with at least 50 stomata analyzed). ns, no significant difference; student’s *t*-test. **(D–I)** Stomatal complex length **(D)**, stomatal complex width **(E)**, stomatal complex ratio (width: length) **(F)**, stomatal pore length **(G)**, pore width **(H)**, and pore ratio (width: length) **(I)** of Col-0, *DcaGDSL47 OE3*, and *DcaGDSL47 OE5*. Data are means ± standard error (n = 5, each with at least 30 stomata measured). *p < 0.05; **p < 0.01; ***p < 0.001; ns, no significant difference; Ordinary one-way ANOVA with Bonferroni’s multiple comparisons test. The measurement of stomatal pore length and width, and stomatal complex length and width was indicated in **(D, E)**.

DcaGDSL47, as a guard cell-enriched GDSL lipase associated with 24 lipid species (Zhan et al., 2022), was investigated for its role in stomatal OCL formation. In *DcaGDSL47-*overexpressing *Arabidopsis*, SEM analyses revealed that an average of 71.63% and 66.46% of the mature stomata in the *DcaGDSL47 OE3* and *DcaGDSL47 OE5* leaves, respectively, had partially degraded stomatal OCL (Type IV), while in Col-0, only 18.54% of the stomata had partially degraded stomatal OCL (**Figures 9A, B**). The ratio of normal opened stomata (Type I) in *DcaGDSL47 OE3* and *DcaGDSL47 OE5* was 22.16% and 20.93%, respectively, significantly lower than the 60. 9% in Col-0, with no substantial alterations in partially occluded stomata (Type II) and fully covered stomata (Type III) (**Figure 9B**). However, stomatal density in *DcaGDSL47-*overexpressing *Arabidopsis* was similar to that in Col-0 (**Figure 9C**), and no substantial alterations were observed in the stomatal complex length, width, and complex ratio (width: length) in Col-0 and *DcaGDSL47-* overexpressing lines (**Figures 9D–F**). Notably, stomatal pore width (width between stomatal OCL) in *DcaGDSL47*-overexpressing lines was significantly wider than that in Col-0 (**Figure 9H**); however, stomatal pore length and stomatal pore ratio were not significantly different between Col-0 and *DcaGDSL47-* overexpressing lines (**Figures 9G, I**). Thus, *DcaGDSL47* compromises drought tolerance by modulating stomatal OCL degradation and increasing stomatal pore width, underscoring its crucial role in stomatal dynamics and drought adaptation in *D. catenatum*.

In summary, aberrant stomatal OCL formation is prevalent across *Dendrobium* species, with drought stress substantially increasing the frequency of Type III stomata (fused stomatal OCL) in *D. catenatum*. Among the 58 identified *GDSL* lipase genes, *DcaGDSL25*, *39*, *47*, and *52* contributed to the regulation of drought tolerance. *DcaGDSL47* (stomata-enriched, ER-localized) exhibited drought-repressed expression, and its heterologous overexpression in *Arabidopsis* decreased drought tolerance, accelerated stomatal water loss, and caused stomatal OCL degradation, indicating that it regulates stomatal OCL formation and drought adaptation. These results underscore the critical role of the GDSL lipase family in stomatal OCL formation and drought adaptation mechanisms in *D. catenatum*.

## 4 Discussion

*D. catenatum*, a perennial epiphytic orchid (*Orchidaceae*), is used as a traditional Chinese medicinal herb. Its bioactive compounds have diverse pharmacological properties, including hypoglycemic, immunomodulatory, antioxidant, anti-aging, and antitumor activities (Xu et al., 2022; Wei et al., 2024). *D. catenatum*, which is naturally adapted to a chronically arid environment, has evolved drought-adaptive traits such as water-storing pseudobulbs, thickened leaves, and a facultative CAM photosynthetic pathway with high water-use efficiency (Zhang et al., 2014; Wan et al., 2018). Its drought resistance mechanisms remain poorly characterized despite it being a naturally selected drought-tolerant species. Stomatal OCL formation is closely linked to compositional or structural modifications of the epidermal wax, cutin, and cell walls, thereby serving as a critical checkpoint for regulating stomatal and epidermal transpiration (Tang et al., 2020) and playing a crucial role in plant drought tolerance (Schnurr et al., 2004; Li et al., 2007; Macgregor et al., 2008; Lu et al., 2012; Hunt et al., 2017; Tang et al., 2020). Our findings indicate prevalent stomatal OCL abnormalities in *D. catenatum*, such as partially occluded stomata with fused cuticular layers, across six Chinese regions (Anlong, Chishui, and Qianxinan in the Guizhou Province; Yulin Rongxian in Guangxi; Zhangzhou in Fujian; and Honghe in Yunnan). Three types of stomata (Type I, II, and III) exist, each with distinct distributions. In Anlong, Chishui, Qianxinan, Yulin, Zhangzhou, and Honghe, Type III stomata (fully occluded stomata) were observed at frequencies of 12, 8, 22, 20, 14, and 27%, respectively. Among them, Honghe (Yunnan) specimens showed the highest frequency of Type III stomata (27%). Furthermore, Type III stomata were also observed in other *Dendrobium* genus, including *D. nobile* (14%), *D. huoshanense* (8%), and *D. fimbriatum* (59%), with *D. fimbriatum* and *D. huoshanense* exhibiting the highest and lowest frequencies, respectively (**Figure 1**). Notably, *Dendrobium genus* exhibited a substantially higher frequency of Type III stomata [mean, 17%] than *Arabidopsis* [2%] (Tang et al., 2020). Thus, aberrant OCL formation is a conserved drought-adaptation strategy in the *Dendrobium* genus, with interspecific variation in stomatal type frequency potentially underlying the differential drought resistance.

Previously, drought stress has been shown to enhance cuticular wax deposition and leaf cutinization as adaptive responses to aridity (Shepherd and Wynne Griffiths, 2006; Lee and Suh, 2022; Liu et al., 2022). We consistently observed that drought treatment substantially increased the proportion of stomata with aberrant stomatal OCL formation (Type II and Type III) and reduced the number of normally formed OCL stomata (Type I) in *D. catenatum* (**Figure 1K**). These findings suggest that drought induces stomatal pore occlusion through the formation of fused cuticular ledges, thereby restricting stomatal conductance and transpiration rates. This adaptive mechanism enhances drought tolerance by minimizing water loss, thereby enabling *D. catenatum* to survival under conditions of water deficit. Consequently, drought-triggered stomatal occlusion is a critical morphological adaptation of this species. However, the precise relationship between stomatal OCL abnormalities and the concomitant cuticular compositional and structural modifications requires further investigation.

The GDSL lipase family, a subclass of the lipolytic enzymes, is characterized by four conserved domains (Blocks I–IV), with four invariant catalytic residues, Ser (I), Gly (II), Asn (III), and His (IV), which are collectively termed SGNH hydrolases (Akoh et al., 2004; Cenci et al., 2022). The N-terminal GDSL motif in the conserved Block I contains a catalytic serine residue that acts as a proton donor at the active site (Akoh et al., 2004). GDSL lipases comprise catalytic and substrate-binding regions, and the structural plasticity of the substrate-binding pocket confers functional versatility, allowing for diverse substrate specificities and biological roles. In plants, GDSL lipases comprise large gene families that play a crucial role in growth, development, and stress responses (Shen et al., 2022). Genome-wide analyses have identified 105 members in *Arabidopsis* (Lai et al., 2017), 114 in *rice* (Chepyshko et al., 2012), 121 in *Brassica rapa* (Dong et al., 2016), 194 in *soybean* (Su et al., 2020), 103 in *Zea mays* (An et al., 2019), and 198 in *cotton* [*Gossypium hirsutum*] (Liu et al., 2023). While 52 GDSL lipases have been previously reported in *D. catenatum* (Zhan et al., 2022), our identification of 6 additional candidates (DcaGDSL26, 27, 36, 48, 51, and 58) containing the PF00657 domain (Chepyshko et al., 2012) has expanded the family to 58 members (**Figure 2**, **Supplementary Table 2**). However, DcaGDSL lipase activity requires further experimental validation *in vitro* and *in vivo* enzymatic assays.

Multiple plant GDSL lipases regulate drought tolerance via distinct mechanisms. Rice WDL1, an ER-localized GDSL lipase, modulates epidermal cell differentiation and cuticle formation. Furthermore, *wdl1* mutants exhibit smaller stomatal guard cells and pavement cells, defective cuticles, increased transpiration rates, and accelerated water loss (Park et al., 2010). *Arabidopsis* OSP1 lipase, expressed in the leaf epidermis and guard cells, mediates wax biosynthesis and stomatal OCL formation. *osp1* mutants exhibit reduced leaf wax content, approximately 50% Type III stomata, decreased stomatal conductance, and enhanced drought tolerance (Tang et al., 2020). *Soybean* (Glycine max) *GmGELP28* lipase gene is drought-inducible, and its overexpression enhances drought tolerance in *Arabidopsis* and *soybean*, identifying it as a key candidate for drought adaptation (Su et al., 2020). Similarly, the cotton GDSL lipase gene *GhirGDSL26* (Gh_A01G1774) is drought-responsive and confers improved drought resistance (Liu et al., 2023). These results suggest that GDSL lipases regulate drought adaptation through pathways involving cuticle and stomatal development, as well as drought stress induction. In the present study, we analyzed the expression patterns of *DcaGDSLs* in *D. catenatum* across various tissues or under drought stress. Candidate genes that demonstrated high expression in the leaves or drought-responsive induction were selected for heterologous overexpression in *Arabidopsis*. Drought tolerance assays showed that *DcaGDSL25*-, *39*-, *47*-, and *52*-overexpressing lines exhibited decreased drought tolerance (**Figure 7**). These results establish DcaGDSL25, 39, 47, and 52 as potential drought adaptation regulators in *D. catenatum*.

Aberrant stomatal OCL formation critically affects plantranspiration water loss, internal CO_2_ concentration, and photosynthetic efficiency, and plays a crucial role in drought tolerance and photosynthetic product polysaccharide accumulation (Hunt et al., 2017; Tang et al., 2020). In *D. catenatum*, partial stomatal OCL malformation occurs under normal conditions, and drought stress substantially increases its prevalence (**Figure 1**). *D. catenatum*, with its extreme drought tolerance and high stomatal OCL abnormality frequency, serves as an ideal model for stomatal OCL biogenesis research because the molecular mechanisms of stomatal OCL formation remain poorly characterized. Although *Arabidopsis* GDSL lipase OSP1 regulates wax biosynthesis and stomatal OCL formation via thioesterase-mediated VLCFA-CoAs conversion (Tang et al., 2020), *D. catenatum* GDSL lipases involved in this process remain uncharacterized. To address this aspect, we profiled the expression of drought-responsive GDSL lipase candidates (*DcaGDSL25*, *39*, *47*, and *52*) and identified their tissue-specific expression using GUS reporter assays. *DcaGDSL25* and *DcaGDSL52* were strongly expressed in the root tips, leaf veins, trichomes, epidermal cells, mesophyll cells, and guard cells. Contrastingly, *DcaGDSL39* expression was restricted to the root tips, leaf veins, trichomes, and cotyledonary epidermal/mesophyll cells and was absent in true leaves (**Figure 8**). These expression patterns suggest that *DcaGDSL25*, *39*, and *52* may regulate drought tolerance by modulating trichome and vascular development, potentially influencing epidermal and stomatal functions. Notably, *DcaGDSL47* revealed guard cell-enriched expression and drought-suppressed transcription (**Figures 5I2**, **8**), and *DcaGDSL47-*overexpressing *Arabidopsis* decreased drought tolerance, increased stomatal water loss, and caused stomatal OCL degradation (**Figures 7**, **9**). These findings indicate that *DcaGDSL47* is a key regulator of stomatal OCL formation and drought adaptation in *D. catenatum*. However, further studies should characterize stomatal OCL formation and drought tolerance in *DcaGDSL47*-overexpressing or knockout *D. catenatum* plants to define its role. Additionally, DcaGDSL47 (Dca016600) exhibited confirmed lipase activity and positively correlated with 24 lipid species, including Fatty Acyls (FA, WE), Serol lipid (StE, SiE, ChE, ZyE), Sphingolipid (SM, phSM, GD2, CerP), Glycerophospholipid (PIP2, PE, PC, LPI, LPE, LPC, PIP, PI, PG, CL), Glycerolipid (DG), Saccharolipid (MGDG and DGDG), and Prenol lipid (CO). Among these lipids, all except FA showed higher abundance in the leaves of *D. catenatum* compared to roots, stems, and flowers (**Supplementary Figure 4**) (Zhan et al., 2022), suggesting that DcaGDSL47 may modulate stomatal dynamics through the regulation of lipid metabolism in the leaf tissues. Identifying the specific substrate of DcaGDSL47 through integrated lipidomics and *in vitro* enzymatic assays will elucidate the mechanism by which DcaGDSL47 modulates stomatal OCL formation and drought tolerance. However, identifying the specific substrates of DcaGDSL lipases in the stomatal OCL requires prior compositional analysis of the stomatal OCL and systematic comparison with cuticular wax and cutin components, and general leaf lipids. Therefore, elucidating the precise composition of the stomatal OCL represents a key direction for future research. Such efforts would establish a crucial foundation for unraveling the molecular mechanisms underlying stomatal OCL formation.

This study revealed that *D. catenatum* demonstrates a prevalent stomatal OCL malformation that critically contributes to its exceptional drought tolerance in extremely arid environments. GDSL lipases, as a ubiquitous gene family in plants, play crucial roles in growth, development, and adaptation to stress. We identified 58 putative GDSL lipase genes in *D. catenatum*, among which *DcaGDSL25*, *39*, *47*, and *52* were found to functionally regulate drought tolerance. Guard cell-enriched *DcaGDSL47* has been hypothesized to modulate stomatal OCL formation via lipid metabolic reprogramming, thereby governing drought adaptation in *D. catenatum*. These results establish a molecular foundation for understanding drought tolerance mechanisms and stomatal OCL biogenesis in *D. catenatum*, thereby providing novel insights into extreme drought resistance in plants.

## Data Availability

Publicly available datasets were analyzed in this study. This data can be found here: [NCBI database under BioProject PRJNA348403] and [NCBI Sequence Read Archive under SRP132541]. The original contributions presented in the study are included in the article/**Supplementary Material**. Further inquiries should be directed to the corresponding author.

## Supplementary material

Supplementary Figure 1 Heatmap of the *DcaGDSL* gene expression profiles across various tissues.

Supplementary Figure 2 Expression levels of the *DcaGDSL* genes in the transgenic *Arabidopsis* leaves.

Supplementary Figure 3 Water loss rate in the detached leaves of Col-0 and two transgenic lines expressing *35S_pro_: DcaGDSL47-YFP* (*DcaGDSL47 OE3* and *DcaGDSL47 OE5*) in *Arabidopsis* under dark conditions.

Supplementary Figure 4 The abundance of lipid species exhibits a positive correlation with DcaGDSL47 in the roots, stems, leaves, and flowers of *D. catenatum*.

Supplementary Table 1 Primers used in this study.

Supplementary Table 2 Characteristics of the GDSL lipases in *D. catenatum*.

Supplementary Table 3 Expression levels of the *DcaGDSL* genes under extreme drought stress and well-watered conditions.
