# Supplementary material for: Genome-wide identification and characterization of the GDSL lipase gene family in Dendrobium catenatum and their potential role in drought stress tolerance and stomatal outer cuticular ledge formation
Source: Front Plant Sci. 2025 Dec 19;16:1722133. doi: 10.3389/fpls.2025.1722133 (PMC12757301; doi:10.3389/fpls.2025.1722133)
Supplement: Supplementary Figure 1 — Heatmap of the DcaGDSL gene expression profiles across various tissues. [file Table1.docx]

Supplementary Material

# Supplementary Figures and Tables

The following Supplementary Material is available for this article:

**Supplementary Figure 1.** Heatmap of the *DcaGDSL* gene expression profiles across various tissues.

**Supplementary Figure 2.** Expression levels of the *DcaGDSL* genes in the transgenic *Arabidopsis* leaves.

**Supplementary Figure 3.** Water loss rate in the detached leaves of Col-0 and two transgenic lines expressing *35S_pro_:DcaGDSL47-YFP* (*DcaGDSL47 OE3* and *DcaGDSL47 OE5*) in *Arabidopsis* under dark conditions.

**Supplementary Figure 4.** The abundance of lipid species exhibits a positive correlation with DcaGDSL47 in the roots, stems, leaves, and flowers of *D. catenatum*.

**Supplementary Table 1.** Primers used in this study.

**Supplementary Table 2.** Characteristics of GDSL lipases in *D. catenatum*.

**Supplementary Table 3.** Expression levels of the *DcaGDSL* genes under extreme drought stress and well-watered conditions.


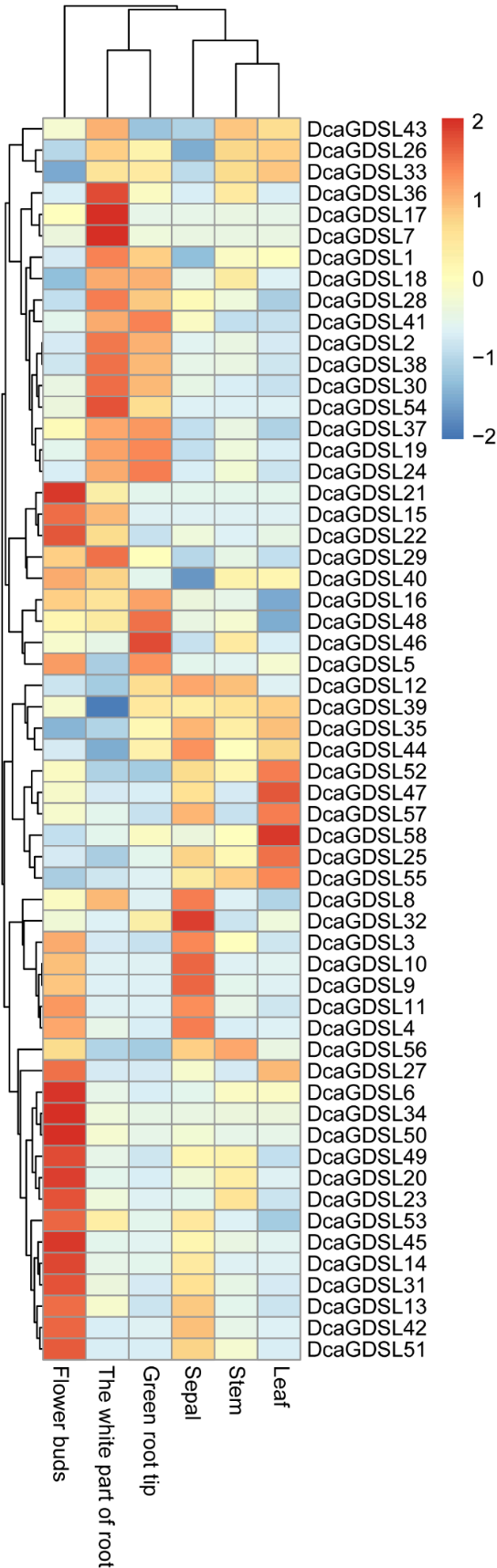


**Supplementary Figure 1.** Heatmap of *DcaGDSL* gene expression profiles across different tissues. FPKM values were normalized with logarithm base two. The scale represents the relative signal intensity of FPKM values.

**
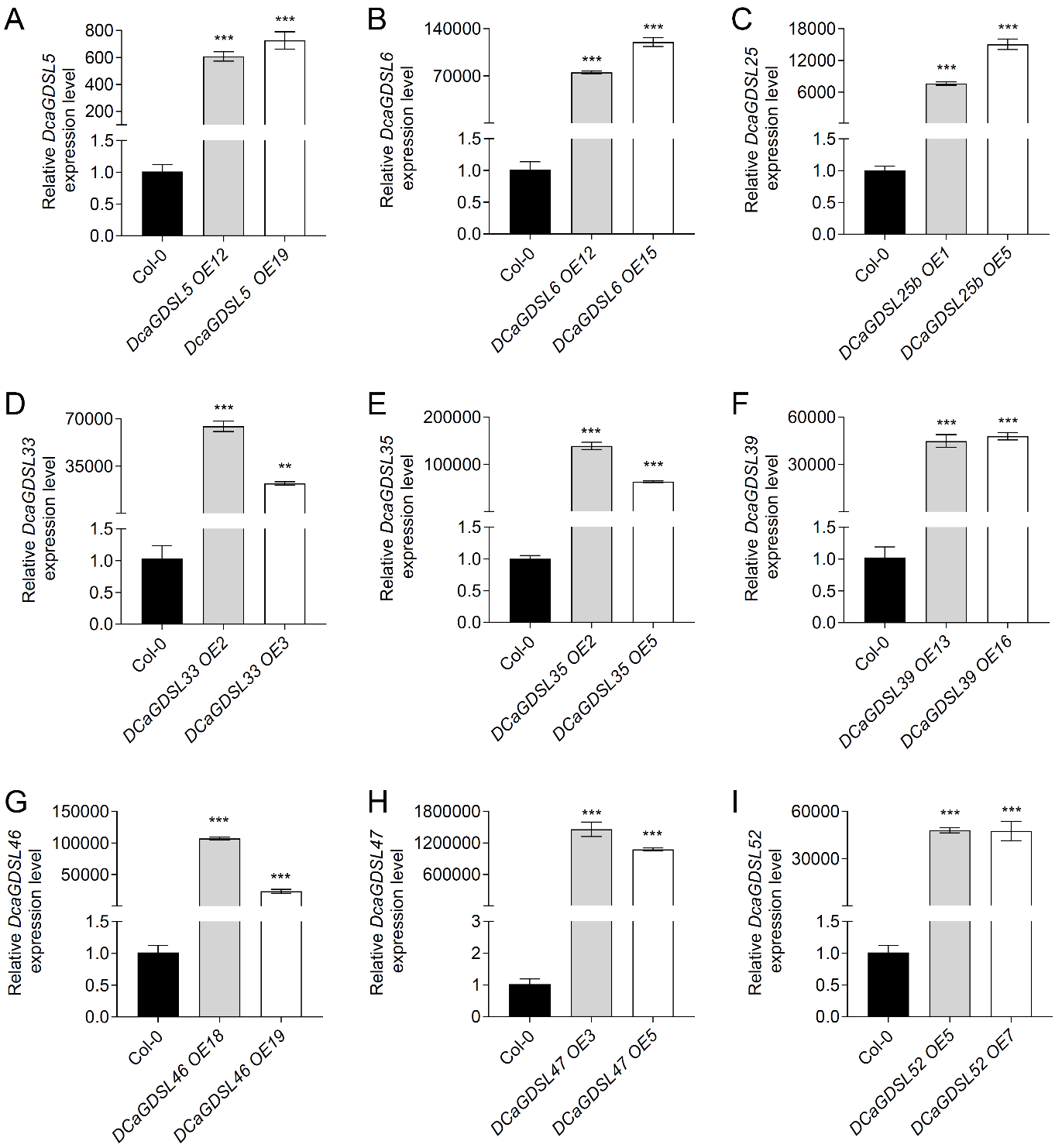
**

**Supplementary Figure 2.** Expression levels of *DcaGDSL* genes in the *DcaGDSLs* transgenic *Arabidopsis* leaves. **(A-I)** Expression level analyses of the *DcaGDSL5* **(A)**, *DcaGDSL6* **(B)**, *DcaGDSL25* **(C)**, *DcaGDSL33* **(D)**, *DcaGDSL35* **(E)**, *DcaGDSL39* **(F)**, *DcaGDSL46* **(G)**, *DcaGDSL47* (H), and *DcaGDSL52* (I) in Col-0 and *DcaGDSLs* transgenic lines relative to *AtNEfa* revealed using qRT-PCR. Relative expression was calculated versus the Col-0 (set as 1.0). Values are represented as means ± standard error (n = 3). **, *P* < 0. 01; ***, *P* < 0. 001; ns, no significant difference; Ordinary one-way ANOVA with Bonferroni's multiple comparisons test.


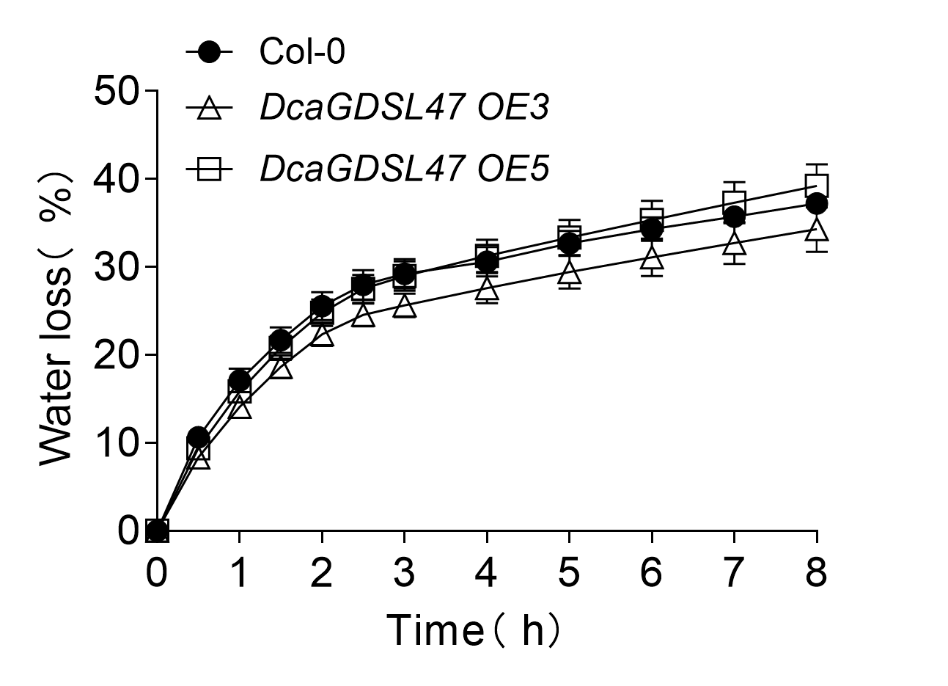


**Supplementary Figure 3.** Water loss rate in the detached leaves of Col-0 and two transgenic lines expressing *35S_pro_:DcaGDSL47-YFP* (*DcaGDSL47 OE3* and *DcaGDSL47 OE5*) in *Arabidopsis* under dark conditions. Values are means ± standard error (n = 3).

**Supplementary Figure 4.** The abundance of lipid species exhibits a positive correlation with DcaGDSL47 in the roots, stems, leaves, and flowers of *D. catenatum* (Zhan et al., 2022).

**References**

Zhan, X., Qian, Y., and Mao, B. (2022). Identification of Two GDSL-Type Esterase/Lipase Genes Related to Tissue-Specific Lipolysis in *Dendrobium catenatum* by Multi-Omics Analysis. Life (Basel) 12(10). doi: 10.3390/life12101563.

**Supplementary Table 1.** Primers used in this study.

| Primer | Sequence（5’-3’） | Usage |
| --- | --- | --- |
| DcaGDSL3-F  DcaGDSL3-R  DcaGDSL5-F  DcaGDSL5-R  DcaGDSL6-F  DcaGDSL6-R  DcaGDSL25-F  DcaGDSL25-R  DcaGDSL33-F  DcaGDSL33-R  DcaGDSL35-F  DcaGDSL35-R  DcaGDSL39-F  DcaGDSL39-R  DcaGDSL46-F  DcaGDSL46-R  DcaGDSL47-F  DcaGDSL47-R  DcaGDSL52-F  DcaGDSL52-R | GGGGACAAGTTTGTACAAAAAAGCAGGCTTAATGGAGGGCAGGCCTATTTC  GGGGACCACTTTGTACAAGAAAGCTGGGTATGCAATAAGGCCAATGCCTTC  GGGGACAAGTTTGTACAAAAAAGCAGGCTTAATGGCTTCTTCCATCACTGG  GGGGACCACTTTGTACAAGAAAGCTGGGTAAAGGGCACTCAACTGCATTAC  GGGGACAAGTTTGTACAAAAAAGCAGGCTTAATGAAACTCACGCTCCTCTC  GGGGACCACTTTGTACAAGAAAGCTGGGTAGATCATAAGAGGGAGATCAG  GGGGACAAGTTTGTACAAAAAAGCAGGCTTAATGAGCTTAAGAAAAGCCG  GGGGACCACTTTGTACAAGAAAGCTGGGTAACATATTGGTTTAAGATTGC  GGGGACAAGTTTGTACAAAAAAGCAGGCTTAATGGCTTCCTCCTCCTTCTC  GGGGACCACTTTGTACAAGAAAGCTGGGTATTCTTCTTTGGAAACATTAATTG  GGGGACAAGTTTGTACAAAAAAGCAGGCTTAATGAATTCACCAATTCAGATTC  GGGGACCACTTTGTACAAGAAAGCTGGGTACAAAGCAAATGCCTCCCAAT  GGGGACAAGTTTGTACAAAAAAGCAGGCTTAATGACTTCCCCTCCTTTTCG  GGGGACCACTTTGTACAAGAAAGCTGGGTAGGCATCATCGACCTTATGATC  GGGGACAAGTTTGTACAAAAAAGCAGGCTTAATGAAGCTGCTCTTCTTTCTTG  GGGGACCACTTTGTACAAGAAAGCTGGGTAATTATTAATAAGCACGTG  GGGGACAAGTTTGTACAAAAAAGCAGGCTTACCAAGGCACAGAAGATAGC  GGGGACCACTTTGTACAAGAAAGCTGGGTAATTTAGTGATGCACCATATTTC  GGGGACAAGTTTGTACAAAAAAGCAGGCTTAATGGCTGCTCTTCTTCTTCTTC  GGGGACCACTTTGTACAAGAAAGCTGGGTAAAGAACACTCAGTTGCCTCAC | Overexpression/ Subcellular localization |
| QDcaGDSL3-F  QDcaGDSL3-R  QDcaGDSL5-F  QDcaGDSL5-R  QDcaGDSL6-F  QDcaGDSL6-R  QDcaGDSL25-F  QDcaGDSL25-R  QDcaGDSL33-F  QDcaGDSL33-R  QDcaGDSL35-F  QDcaGDSL35-R  QDcaGDSL39-F  QDcaGDSL39-R  QDcaGDSL46-F  QDcaGDSL46-R  QDcaGDSL47-F  QDcaGDSL47-R  QDcaGDSL52-F  QDcaGDSL52-R  QDcaActin7-F  QDcaActin7-R  QAtNEfa-F  QAtNEfa-R | GCAAATCCTTCTAAGAGTGGTTTC GCCAATGCCTTCTACGATGAG  CCAGCTGATTATGGATTTACTGAG  AGGGCACTCAACTGCATTACA  AGGCCATTTTGGTTTTGGTCG  GGGAGATCAGTGAAAGAGCCT  CGAAATGAGCCCACAAGATTACC  GTACAACTCCTTAATAGTCCAAGC  GCACAAGGCTTGAAAGAGTACC  ATCAGCAAAAGGCCCTTCCA  CCAGCCAAATCAGGGTTTAAAG  CCGTTCCACAACATATCTGC  TGCTCTACAGATTCAGACTGCA  CAATGTCTTCGCACCAAGTTCG  TATATAGCTTCAATGTTGGGCGTC  GCTCCGGCATTGATATCACC  GCTAGTGTCTGTGATTTCCTTGG  CTTCCTAAATGGTGTGGCGAAG  GATGCCTCAGCTAATGCTG  TTCCCAACCCACAACATGCA  AGTTCCTATTTATGAGGGTTATGC  CCTGACAATTTCACGCTCTG  AATGGTGACGCTGGTATG  CTTCTTGTCCACGCTCTT | qRT-PCR |

**Supplementary Table 2.** Characteristics of GDSL lipases in *D. catenatum*.

| **Gene name** | **Gene ID** | **Locus tag** | **Accession ID** | **Amino acid** | **Molecular**  **weight**  **(kDa)** | **Isoelectric**  **point (pI)** | **Homologous genes in *Arabidopsis*** |
| --- | --- | --- | --- | --- | --- | --- | --- |
| DcaGDSL1 | LOC114578363 | Dca004108 | XP_028549942.1 | 450 | 28.17 | 5.07 | At2g27360-like |
| DcaGDSL2 | LOC110103410 | Dca004419 | XP_020687782.1 | 349 | 38.87 | 5.52 | GDSL esterase/lipase At2g04570 (OSP1) |
| DcaGDSL3 | LOC110105207 | Dca005399 | XP_020690263.1 | 362 | 38.88 | 8.02 | GDSL esterase/lipase APG |
| DcaGDSL4 | LOC110114990 | Dca005843 | XP_028550587.1 | 354 | 39.33 | 6.65 | At3g26430 |
| DcaGDSL5 | LOC110107155 | Dca006717 | XP_020692989.1 | 366 | 30.09 | 6.02 | At5g55050 |
| DcaGDSL6 | LOC110116501 | Dca010247 | XP_020705738.1 | 363 | 40.18 | 7.31 | At5g45910 |
| DcaGDSL7 | LOC110116531 | Dca010248 | XP_028553561.1 | 801 | 86.96 | 4.8 | uncharacterized LOC110116531 |
| DcaGDSL8 | LOC110116532 | Dca010249 | XP_020705795.2 | 361 | 40.10 | 6.87 | GDSL esterase/lipase At5g45910 |
| DcaGDSL9 | LOC110094705 | Dca010377 | XP_020675661.2 | 572 | 63.79 | 8.38 | At3g26430-like |
| DcaGDSL10 | LOC110110649 | Dca010687 | XP_028556994.1 | 282 | 31.14 | 6.49 | At1g71691-like |
| DcaGDSL11 | LOC110110643 | Dca010688 | XP_028556995.1 | 383 | 43.25 | 8.52 | At1g71250 |
| DcaGDSL12 | LOC110098379 | Dca013394 | XP_020680858.1 | 394 | 43.68 | 6.61 | At3g26430 |
| DcaGDSL13 | LOC110097838 | Dca014367 | XP_020680104.1 | 370 | 40.54 | 5.1 | At5g45670 |
| DcaGDSL14a | LOC110111616 | Dca014436 | XP_028555802.1 | 377 | 41.74 | 4.89 | At5g33370-like /LTL1-like |
| DcaGDSL14b |  |  | XP_028555803.1 | 376 | 41.71 | 4.89 |  |
| DcaGDSL14c |  |  | XP_028555804.1 | 364 | 40.46 | 5.01 |  |
| DcaGDSL14d |  |  | XP_028555805.1 | 363 | 40.28 | 4.79 |  |
| DcaGDSL14e |  |  | XP_028555806.1 | 363 | 40.20 | 4.7 |  |
| DcaGDSL14f |  |  | XP_028555807.1 | 363 | 40.18 | 4.7 |  |
| DcaGDSL14g |  |  | XP_028555808.1 | 363 | 40.09 | 4.9 |  |
| DcaGDSL14h |  |  | XP_028555809.1 | 359 | 39.83 | 4.87 |  |
| DcaGDSL15 | LOC110113223 | Dca015397 | XP_020701352.1 | 378 | 41.74 | 5.55 | At1g71691-like |
| DcaGDSL16 | LOC110112873 | Dca016599 | XP_020700914.1 | 363 | 40.16 | 8.35 | EXL3-like |
| DcaGDSL17 | LOC110105379 | Dca020540 | XP_028553658.1 | 338 | 36.91 | 6.42 | At5g55050-like |
| DcaGDSL18 | LOC110116410 | Dca020661 | XP_020705615.1 | 378 | 41.61 | 6.84 | Acetylajmalan esterase |
| DcaGDSL19 | LOC110094371 | Dca023828 | XP_020675249.1 | 373 | 40.98 | 7.73 | At2g23540 |
| DcaGDSL20 | LOC110115107 | Dca024181 | XP_020703903.1 | 376 | 41.14 | 6.9 | At1g71691 |
| DcaGDSL21 | LOC110115108 | Dca024182 | XP_020703904.1 | 366 | 41.09 | 7.06 | At1g33811 |
| DcaGDSL22 | LOC110109094 | Dca025482 | XP_020695663.1 | 362 | 40.09 | 5 | At5g45960 |
| DcaGDSL23 | LOC110092180 | Dca008857 | XP_020672263.1 | 371 | 40.79 | 9.78 | At1g29670 |
| DcaGDSL24 | LOC110094172 | Dca026195 | XP_020674995.1 | 355 | 39.36 | 6.24 | At5g37690 |
| DcaGDSL25a | LOC110095430 | Dca026097 | XP_020676630.1 | 385 | 42.19 | 6.84 | At4g01130-like |
| DcaGDSL25b |  |  | XP_028549044.1 | 335 | 36.57 | 8.26 |  |
| DcaGDSL26 | LOC110097456 | -- | XP_020679489.1 | 329 | 36.10 | 7.67 | At2g27360-like |
| DcaGDSL27 | LOC110097548 | -- | XP_020679643.1 | 346 | 38.08 | 4.74 | At1g71250 |
| DcaGDSL28 | LOC110098250 | Dca006411 | XP_020680666.1 | 369 | 40.13 | 5.16 | At2g23540 |
| DcaGDSL29 | LOC110099105 | Dca010143 | XP_020681810.1 | 365 | 39.44 | 8.38 | At5g55050 |
| DcaGDSL30 | LOC110103964 | Dca012485 | XP_020688530.1 | 360 | 38.99 | 5.3 | At2g23540 |
| DcaGDSL31 | LOC110104384 | Dca002293 | XP_020689123.1 | 380 | 41.38 | 4.62 | At5g33370 |
| DcaGDSL32a | LOC110104937 | Dca014323 | XP_020689908.1 | 386 | 42.31 | 7.22 | At4g01130 |
| DcaGDSL32b |  |  | XP_020689909.1 | 321 | 35.16 | 6.41 |  |
| DcaGDSL33 | LOC110106209 | Dca004110 | XP_020691667.1 | 391 | 42.91 | 5.83 | At2g27360-like |
| DcaGDSL34 | LOC110106260 | Dca018070 | XP_020691749.1 | 366 | 40.44 | 5.75 | GLIP7 |
| DcaGDSL35 | LOC110107193 | Dca019638 | XP_020693039.1 | 390 | 42.60 | 4.53 | GLIP5 |
| DcaGDSL36 | LOC110107345 | -- | XP_028547857.1 | 154 | 17.50 | 8.3 | At5g45910-like |
| DcaGDSL37a | LOC110107731 | Dca011690 | XP_020693750.1 | 384 | 41.97 | 7.04 | At1g54790 |
| DcaGDSL37b |  |  | XP_028550429.1 | 317 | 34.87 | 6.98 |  |
| DcaGDSL38 | LOC110107863 | Dca004940 | XP_020693954.1 | 361 | 39.98 | 7.69 | At1g74460 |
| DcaGDSL39 | LOC110108246 | Dca010424 | XP_020694466.1 | 394 | 43.20 | 5.33 | At1g28570 |
| DcaGDSL40 | LOC110109902 | Dca022149 | XP_020696775.1 | 375 | 40.99 | 4.89 | LIP-4 |
| DcaGDSL41 | LOC110110493 | Dca012005 | XP_020697656.1 | 358 | 39.62 | 9.26 | At1g74460 |
| DcaGDSL42 | LOC110111803 | Dca000029 | XP_020699480.1 | 373 | 41.14 | 7.77 | At3g48460 |
| DcaGDSL43 | LOC110112168 | Dca018199 | XP_020699937.1 | 367 | 40.22 | 8.17 | EXL3 |
| DcaGDSL44 | LOC110112668 | Dca011126 | XP_020700626.1 | 391 | 42.76 | 6.41 | At4g01130-like |
| DcaGDSL45 | LOC110112861 | Dca016598 | XP_020700898.1 | 364 | 40.26 | 8.02 | EXL3 |
| DcaGDSL46 | LOC110112864 | Dca016597 | XP_020700901.1 | 358 | 39.24 | 5.72 | EXL3 |
| DcaGDSL47 | LOC110112874 | Dca016600 | XP_020700915.1 | 362 | 39.68 | 8.36 | EXL3 |
| DcaGDSL48 | LOC110112875 | -- | XP_028547457.1 | 399 | 44.53 | 8.37 | EXL3-like |
| DcaGDSL49 | LOC110112945 | Dca013263 | XP_020701009.1 | 354 | 37.66 | 7.24 | At5g03810 |
| DcaGDSL50 | LOC110113970 | Dca022896 | XP_020702355.1 | 367 | 40.16 | 8.25 | At5g33370 |
| DcaGDSL51 | LOC110115002 | -- | XP_020703746.2 | 221 | 24.82 | 9.77 | Esterase-like |
| DcaGDSL52 | LOC110115925 | Dca010602 | XP_020704993.1 | 353 | 37.79 | 7.41 | At5g55050-like |
| DcaGDSL53 | LOC110116640 | Dca023731 | XP_020705963.1 | 354 | 39.49 | 6.59 | At4g26790 |
| DcaGDSL54 | LOC114579599 | Dca005844 | XP_028550588.1 | 381 | 42.03 | 9.45 | At3g26430-like |
| DcaGDSL55 | LOC110094859 | Dca022174 | XP_020675851.1 | 238 | 26.32 | 4.84 | At5g45920 |
| DcaGDSL56 | LOC110108775 | Dca004347 | XP_020695215.1 | 291 | 33.13 | 8.89 | At5g62930 |
| DcaGDSL57a | LOC110092624 | Dca026099 | XP_020672922.1 | 164 | 17.57 | 6.71 | At4g01130-like |
| DcaGDSL57b |  |  | XP_028549045.1 | 162 | 17.47 | 5.52 |  |
| DcaGDSL58 | LOC110108244 | -- | XP_028548571.1 | 357 | 38.95 | 5.58 | GDSL esterase/lipase At1g28590-like |

**Supplementary Table 3.** Expression levels of *DcaGDSL* genes under extreme drought stress and well-watered conditions.

| **Name** | **Gene ID** | **Well_watered (ww) Expression** | | **Extreme_drought (ED) Expression** | **Log_2_ Fold Change** | | | | **P-value** | **ED vs WW**  **Up/Down** | | |
| --- | --- | --- | --- | --- | --- | --- | --- | --- | --- | --- | --- | --- |
| DcaGDSL3 | LOC110105207 | 122.14 | 7.00 | | | -4.13 | 9.40E-05 | | | | Down |  |
| DcaGDSL5 | LOC110107155 | 20.62 | 2.51 | | | -3.04 | | 0.006179904 | | | Down |  |
| DcaGDSL6 | LOC110116501 | 50.57 | 12.45 | | | -2.02 | | 0.000527334 | | | Down |  |
| DcaGDSL39 | LOC110108246 | 2945.85 | 1464.82 | | | -1.01 | | 4.70E-05 | | | Down |  |
| DcaGDSL46 | LOC110112864 | 16.32 | 64.45 | | | 1.98 | | 0.00026558 | | | Up |  |
| DcaGDSL52 | LOC110115925 | 134.77 | 23.75 | | | -2.50 | | 7.13E-21 | | | Down |  |
